# The Roles of Left Versus Right Anterior Temporal Lobes in Conceptual Knowledge: An ALE Meta-analysis of 97 Functional Neuroimaging Studies

**DOI:** 10.1093/cercor/bhv024

**Published:** 2015-03-13

**Authors:** Grace E. Rice, Matthew A. Lambon Ralph, Paul Hoffman

**Affiliations:** Neuroscience and Aphasia Research Unit (NARU), School of Psychological Sciences, University of Manchester, Manchester, UK

**Keywords:** ALE meta-analysis, anterior temporal lobes, conceptual knowledge, laterality, semantic memory

## Abstract

The roles of the right and left anterior temporal lobes (ATLs) in conceptual knowledge are a source of debate between 4 conflicting accounts. Possible ATL specializations include: (1) Processing of verbal versus non-verbal inputs; (2) the involvement of word retrieval; and (3) the social content of the stimuli. Conversely, the “hub-and-spoke” account holds that both ATLs form a bilateral functionally unified system. Using activation likelihood estimation (ALE) to compare the probability of left and right ATL activation, we analyzed 97 functional neuroimaging studies of conceptual knowledge, organized according to the predictions of the three specialized hypotheses. The primary result was that ATL activation was predominately bilateral and highly overlapping for all stimulus types. Secondary to this bilateral representation, there were subtle gradations both *between* and *within* the ATLs. Activations were more likely to be left lateralized when the input was a written word or when word retrieval was required. These data are best accommodated by a *graded* version of the hub-and-spoke account, whereby representation of conceptual knowledge is supported through bilateral yet graded connectivity between the ATLs and various modality-specific sensory, motor, and limbic cortices.

## Introduction

Convergent evidence has implicated the bilateral anterior temporal lobes (ATLs), as critical neural substrates for the semantic representation of words, objects, people, and social concepts (Sergent et al. 1992; Marinkovic et al. 2003; Olson et al. 2007; Patterson et al. 2007; Pobric et al. 2007; Lambon Ralph, Sage, et al. 2010; Visser, Embleton, et al. 2010; Lambon Ralph 2014). Research attention has now shifted to exploring how conceptual knowledge is represented *within* this bilateral system, with a particular focus on the functions of the right and left ATLs (Olson et al. 2007; Pobric et al. 2007; Lambon Ralph et al. 2009; Pobric et al. 2010; Visser, Jefferies, et al. 2010; Drane et al. 2013; Gainotti 2012, 2013; Wong and Gallate 2012; Olson et al. 2013). This large-scale meta-analysis focused on this issue and formally evaluated four prominent accounts from the literature. The “ATL hub-and-spoke” account proposes that the right and left ATLs represent conceptual knowledge in a unified manner as part of a bilateral, coupled system [thereby promoting robust representations: see Schapiro et al. (2013)]. An extreme version of this account would predict no differences between the hemispheres; however, a more nuanced position holds that graded hemispheric specialization emerges as a consequence of differential connectivity (Lambon Ralph et al. 2001; Binney et al. 2012; Schapiro et al. 2013). Conversely, a greater degree of specialization between the right and left ATLs has been proposed, reflecting (1) the modality of stimulus input (Gainotti 2007, 2013), (2) the involvement of word retrieval or visual recognition in the task (Damasio et al. 2004), or (3) the social content of the stimulus (Olson et al. 2007; Zahn et al. 2007). The development of these four accounts has largely been in parallel. There is now a large body of published functional neuroimaging data that can be used to evaluate each of these theories. The key aims of this large-scale meta-analysis were, therefore, to provide a novel synthesis of the functional imaging literature in healthy individuals and a direct simultaneous evaluation of the differing accounts. The principal features of each hypothesis are briefly described below.

We and others have proposed that the right and left ATLs work in tandem as a bilateral, partially redundant system (“ATL hub-and-spoke”: Patterson et al. 2007; Lambon Ralph, Cipolotti, et al. 2010; Lambon Ralph et al. 2012; Schapiro et al. 2013; Lambon Ralph 2014). Interest in the ATLs as a conceptual region primarily stems from the study of semantic dementia (SD) patients who exhibit a selective yet progressive multimodal impairment of conceptual knowledge (Hodges et al. 1992; Bozeat et al. 2000; Snowden et al. 2004; Jefferies et al. 2009). In this patient group, atrophy is always bilateral (though often asymmetric; Hodges et al. 1992; Lambon Ralph et al. 2001). Performance on semantic tasks in SD patients is correlated with the amount of atrophy and hypometabolism in both ATLs, centered on the ventrolateral surface (Butler et al. 2009; Mion et al. 2010), which directly mirrors findings from neurologically intact participants in PET or distortion-corrected fMRI studies (Sharp et al. 2004; Binney et al. 2010; Visser and Lambon Ralph 2011). Unilateral damage to the same areas has much milder effects on semantic performance (Lambon Ralph, Cipolotti, et al. 2010; Bi et al. 2011), which can be detected only if sensitive semantic tasks are utilized (Lambon Ralph et al. 2012). This suggests that the semantic system can withstand the effects of unilateral damage more successfully than an equivalent amount of bilateral damage—the same conclusion as drawn from seminal primate studies of unilateral versus bilateral ATL resection (Brown and Schafer 1888; Kluver and Bucy 1937, 1939). Schapiro et al. (2013) demonstrated this basic principle in a computational model and formal mathematical analysis which incorporated the assumption that the ATLs represent conceptual knowledge, bilaterally. In an extension of the Rogers et al. (2004) hub-and-spoke computational model, the “semantic” hidden units in the model were divided into right and left “demi-hubs” (representing the right and left ATLs). When only one of the demi-hubs was damaged, the model's performance was only mildly compromised; when both demi-hubs were damaged, however, the model's semantic performance was severely impaired. Critically, this result held even when the total amount of damage was equated in the unilateral and bilateral lesions (Schapiro et al. 2013). Although primarily a bilateral model of semantic representation, importantly, this account does not preclude graded specialization in each demi-hub. Indeed, in two previous computational models (Lambon Ralph et al. 2001; Schapiro et al. 2013), the left demi-hub was more strongly connected to speech output representations, with the consequence that damage to the left (in comparison with right) demi-hub produced more substantial deficits in picture naming despite equivalent levels of semantic impairment overall.

Other researchers have proposed a more specialized organization of conceptual knowledge in left and right ATLs, based on performance differences between patients with left and right ATL damage (Olson et al. 2007; Acres et al. 2009; Gainotti 2013). The “input modality” account emphasizes differences in ATL function based on the input modality of the task. Accordingly, verbal inputs (written or spoken words) are predicted to be associated with the left ATL and non-verbal inputs (pictures) with the right ATL (Snowden et al. 2004; Gainotti 2007; Snowden et al. 2012). Evidence for this standpoint stems from SD patients, with bilateral yet *asymmetric* ATL damage. Snowden et al. (2004) directly compared the performance of SD patients with R > L or L > R ATL damage on famous face versus written name recognition tasks. Performance on both tasks were impaired in R > L and L > R patient groups, compared with an older adult control group, and exhibited strong item association across face and name modalities (consistent with a bilateral model of semantic representation). However, the L > R group performed more poorly on the name recognition task relative to the R > L group, whereas the R > L group performed more poorly on the face recognition task relative to the L > R group. Similar conclusions have been drawn from studies that used voxel-based morphometry to relate behavioral performance on semantic tasks to the integrity of the ATL gray matter. For example, Butler et al. (2009) studied patients with progressive language deficits from mixed etiologies and correlated their performance on word and picture-based semantic tasks with the degree of damage in each voxel. Damage to both ATLs was negatively correlated with performance on both versions of the semantic task. In addition, damage to the left ATL was more strongly correlated with performance on the word-based version and right ATL damage with performance on the picture-based task.

The word retrieval/visual recognition account predicts differences based on whether the task requires generation of a word based on semantic knowledge or access to semantic knowledge from the visual input (e.g., face recognition). On this view, word retrieval tasks (e.g., naming pictures) rely on the left ATL and other tasks (e.g., object recognition) are supported by the right ATL (Damasio et al. 2004). This approach is again based on performance differences between patients with left and right ATL damage or resection (Tranel et al. 1997; Damasio et al. 2004; Drane et al. 2013). Acres et al. (2009) correlated patients' performance on naming and object recognition with voxel-based morphometry measures of temporal lobe integrity. Damage to the left ATL was correlated with scores on naming tasks, and damage to the right ATL was correlated with scores on visual recognition. Similarly, patients with unilateral lesions to the left ATL exhibit more severe naming deficits relative to patients with unilateral right ATL lesions (Glosser et al. 2003; Drane et al. 2008, 2013; Mesulam et al. 2013). While it is possible to account for these data under the bilateral hub-and-spoke account (see above), the alternative account proposed by these researchers suggests that the left ATL is specialized for the process of lexical access from semantic knowledge, whereas the right ATL is specialized for visual recognition (Damasio et al. 2004; Drane et al. 2013).

The third account suggests either that both ATLs are specialized for coding social concepts or that the right and left ATLs are differentially involved. Many researchers have noted that the ATLs are involved in social cognition in humans and primates (Kluver and Bucy 1937; Edwards-Lee et al. 1997; Frith and Frith 2003; Gallate et al. 2011). More recently, several research groups have proposed that part or all of the ATL codes social concepts, including person knowledge and emotional concepts (Thompson et al. 2003; Olson et al. 2007; Zahn et al. 2007, 2009; Ross and Olson 2010; Olson et al. 2013). Deficits in social behavior are often observed in SD patients, including social awkwardness, person recognition deficits, and a loss of empathy (Thompson et al. 2003; Chan et al. 2009). The current literature is inconsistent with regard to any laterality of social concepts across left versus right ATL regions. Clinically, it has been argued that the social impairments in SD patients are typically more severe, or more obvious, when atrophy is R > L (Edwards-Lee et al. 1997; Miller et al. 1997; Chan et al. 2009; Zahn et al. 2009). In a novel extension from these clinical findings to fMRI, Zahn et al. (2007) demonstrated that activation associated with processing socially related words (e.g., “polite”) versus non-social words (e.g., “nutritious”) was localized to the right anterior superior temporal gyrus (STG). However, more recent fMRI studies of social processing and a direct replication of the Zahn et al. task found greater left than right ATL activations (Skipper et al. 2011; Ross and Olson 2012). Indeed, the potential role of left as well as right ATL in social concepts is emphasized by the study of Chan et al. (2009), which, in a formal exploration, found social and behavioral deficits in L > R and R > L SD patients.

Related to these results for social concepts, laterality effects have sometimes been reported for processing of faces and people's names. Specifically, it has been proposed that the left ATL is preferentially involved in processing names of people and the right ATL is preferentially involved in processing familiar faces (Damasio et al. 2004; Snowden et al. 2004; Gainotti 2013). There is also an ongoing debate as to whether the ATLs preferentially process semantic knowledge for familiar people (Simmons et al. 2010; Von Der Heide, Skipper, Olson 2013), which could be related to their high intrinsic social relevance.

All four accounts described above draw heavily on evidence from patients with ATL damage. While patient studies have provided important insights into ATL function, there are limitations in their ability to distinguish between the roles of left and right ATL. Much of the evidence comes from SD patients, but these patients always have some degree of bilateral atrophy (Galton et al. 2001; Snowden et al. 2004), precluding strong inferences about the function of each ATL. Functional neuroimaging studies in healthy participants provide an important additional source of constraint over the theories discussed above. While individual fMRI and PET studies provide support for each theory, when put side-by-side, a rather mixed picture emerges. For example, ATL activation is often left lateralized (L > R) following presentation of verbal information (Mummery et al. 1999; Marinkovic et al. 2003; Spitsyna et al. 2006; Visser and Lambon Ralph 2011), but a corresponding R > L ATL activation for non-verbal inputs is not found, as ATL activation to these is typically bilateral (Visser and Lambon Ralph 2011; Visser et al. 2012). Similarly, PET studies suggest a left ATL bias for word retrieval tasks (Tranel et al. 1997; Grabowski et al. 2001; Damasio et al. 2004), but non-verbal semantic decisions tend to elicit bilateral activation (Tsukiura et al. 2006; Tsukiura et al. 2008). Likewise, as noted above, neuroimaging evidence is also inconsistent with respect to the involvement of left versus right ATLs in social concepts (Zahn et al. 2007; Ross and Olson 2010).

Rather than picking individual studies, however, the neuroimaging literature on semantic processing is now sufficiently large that formal meta-analytic techniques can be applied to extract reliable trends and used to test the principal ideas from the four contrasting theories, directly. Specifically, the goal of the present study was to aggregate and analyze data from 97 neuroimaging studies of conceptual knowledge. We used activation likelihood estimation (ALE) analysis (Laird et al. 2005), a method that extracts coordinates from a set of neuroimaging studies and estimates the likelihood of activation across each voxel in the brain. The resultant “activation likelihood maps” can then be viewed on a standard brain (Laird et al. 2005). ALE also allows for formal subtractions between two maps to explore differences between conditions and to explore laterality effects (Turkeltaub and Coslett 2010). To compare the bilateral “ATL hub-and-spoke” and the three more specialized accounts of ATL function without any a priori assumptions, three analyses were designed to test the principal notions that differentiate the various hypotheses. With some inevitable simplifications, these map directly onto specific theories that have been articulated in the literature. In addition, they can be tested using a meta-analytic approach. In doing so, of course, the test analyses may not be entirely consistent with every aspect of each specific proposal in the literature, but they do allow a formal mapping and testing of the key hypotheses which, in turn, will inform more sophisticated explorations in future, targeted work.

## Methods

### Study Selection

To explore right and left ATL function in conceptual knowledge, two separate literature searches were undertaken (the full list of studies are listed in Supplementary Table 1). First, 48 studies from a recent meta-analysis investigating the role of the ATLs in conceptual knowledge were re-analyzed (Visser, Jefferies, et al. 2010). The inclusion criteria were: (1) PET or fMRI studies exploring semantic memory, (2) studies reported at least one peak in the ATLs (see below for definition); and (3) studies were published in peer-reviewed journals, in English, between January 1992 and March 2008. The exclusion criteria were: (1) studies which did not explicitly include the ATLs in acquisition or data analysis, (2) studies which focused on individual differences (e.g., sex or age differences), patients, task switching, priming or adaptation, language development, syntax, metaphoric or idiom comprehension, and bilingualism or working/episodic memory demands.

A second literature search was conducted to investigate the role of the ATLs in “social semantics”, as this was not the focus of Visser, Jefferies, et al. (2010). Studies were defined as socially semantic if they conformed to one of three subcategories: (1) *social concepts*: Probing knowledge of socially relevant words or situations (e.g., Zahn et al. 2007; Skipper et al. 2011), (2) *person knowledge*: Person identification through faces, names, or voices (e.g., Sergent et al. 1992; Gorno-Tempini and Price 2001; Bethmann et al. 2012), and (3) *emotion processing*: Eliciting the concept of an emotion (e.g., Reiman et al. 1997). The emotion subcategory was included to test for any differences between emotional versus social content in ATL function. The following search terms were entered into the Web of Knowledge (www.isiknowledge.com): [“fMRI” OR “PET”], [“anterior temporal lobes” OR “ATLs”] combined with [“social concepts,” “person recognition,” “face recognition,” “name recognition,” “voice recognition,” “emotion”]. The inclusion and exclusion criteria were matched to Visser, Jefferies, et al. (2010) with the following amendments: (1) PET or fMRI studies which conformed to one of the three subcategories (social concepts, person knowledge, and emotion concepts), (2) published in peer-reviewed journals, in English, between January 1992 and September 2012. These criteria resulted in a cohort of 49 studies.

### ATL Definition

**Figure 1.**
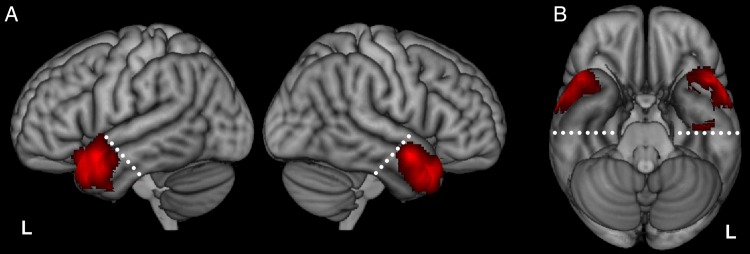
Activation likelihood map showing all 271 foci from 97 studies of conceptual knowledge, shown on the lateral (*A*) and ventral views (*B*). White dashed lines indicate the ATL cutoff; temporal voxels anterior to this plane were defined as falling within the ATL.

ATL is a general term that has been used in slightly different ways by different researchers. Some have used the term in an inclusive fashion to describe areas subsumed by the typical site of atrophy in SD patients (Mion et al. 2010), including the temporal pole and the anterior portions of all temporal gyri (Binney et al. 2010). Others have used the term more selectively to specify temporal cortex anterior to the limen insula (Simmons et al. 2010), anterior to the line *y* = 0 in the MNI space (Visser, Jefferies, et al. 2010), or to include only tissue falling within the temporal pole proper (Tsapkini et al. 2011). These latter definitions tend to exclude the tissue of the anterior inferior temporal gyrus (ITG) and anterior fusiform gyri, which recent fMRI and SD studies have shown to be critical in semantic cognition (Binney et al. 2010; Mion et al. 2010; Visser, Embleton, et al. 2010; Visser et al. 2012). In the context of this study, we wished to ensure that we included the anterior aspects of all the temporal gyri in equal amounts, so we constructed a plane perpendicular to the long axis of the temporal lobe (passing through the fusiform gyrus at *y* = −20, *z* = −30 and STG at *y* = 0, *z* = −5) and defined all temporal peaks anterior to this plane as falling within the ATL (Fig. 1). This region encompassed the temporal pole (BA 38) and anterior portions of the STG (BA 22), middle temporal gyrus (MTG; BA 21), ITG (BA 20), and fusiform gyrus (BA 20).

### Laterality Outside the ATLs

While the main focus of this meta-analysis was exploring ATL function, we also investigated the pattern of laterality in the rest of the brain. To do this, a separate analysis was conducted using all contrast coordinates without the ATL restriction. It is important to note that this analysis was based on the same 97 studies used for the ATL analysis. This means that it only contains studies that reported activations in both ATL and other regions. Investigations that reported non-ATL activations alone were not included in this analysis (which might shift the pattern of non-ATL activation likelihoods reported in this study).

### Study Definition

The cohort of studies was considered in three separate analyses, designed to test the predictions of the three specialized accounts. Each analysis divided the cohort into two different types of study proposed to be related to ATL lateralization. The number of studies in each analysis is listed in Table 1 below. Studies included in each analysis are listed in Supplementary Table 1.

**Table 1:** Number of studies inputted into each analysis

Study type	ATL peaks	Experiments	Subjects
Input modality			
Verbal input	146	53	738
*Auditory words*	*71*	*21*	*241*
*Written words*	*74*	*33*	*528*
Non-Verbal input	113	41	614
Word retrieval			
Word retrieval tasks	50	21	358
*Naming*	*27*	*10*	*195*
*Reading*	23	*11*	*163*
“Other” tasks	217	76	1005
Semantic category			
Non-social semantics	134	48	643
Social semantics	137	49	703
*Social concepts*	*30*	*14*	*183*
*Person knowledge*	*63*	*17*	*285*
*Emotion concepts*	43	*18*	*235*

Note: Subanalyses are shown in italics.

#### Input Modality (Analysis 1)

This analysis compared the probability of right and left ATL activation following “verbal” and “non-verbal” inputs. Studies were considered “verbal” if auditory or written words were presented. Studies were considered “non-verbal” if pictures or non-verbal auditory sounds were presented. Six studies were excluded from this analysis because they could not be classified into either “verbal” or “non-verbal” inputs. To investigate the reported left ATL bias for written information (Marinkovic et al. 2003; Spitsyna et al. 2006; Gainotti 2007), the “verbal input” studies were further subdivided into “auditory words” and “written words.” The “non-verbal input” studies could not be split into “auditory” and “visual” studies because only three studies presented non-verbal auditory stimuli.

#### Word Retrieval (Analysis 2)

This analysis compared the probability of right and left ATL activation during word retrieval versus other tasks. Studies were classified as requiring word retrieval if overt or covert speech generation, such as naming pictures or reading words or sentences, was required. “Other” tasks were those that required some other processing of the stimulus (e.g., lexical decision, semantic judgements, and item classification). One study was excluded from this analysis because no response was required. To investigate the reported left ATL bias for object naming specifically (Tranel et al. 1997; Damasio et al. 2004), the “word retrieval” studies were subdivided into “picture naming” and “word reading” studies.

#### Semantic Category (Analysis 3)

This analysis compared the probability of right and left ATL activation for “non-social semantic” and “social semantic” categories. “Non-social semantic” studies were taken from Visser, Jefferies, et al. (2010) and included studies that investigated general semantic stimuli (e.g., tools, animals). “Social semantic” studies were taken from the second literature search and included studies which required judgements about socially relevant words, people, or emotions. To explore the hypothesized right ATL social bias (Miller et al. 1997; Olson et al. 2007), the “social semantic” studies were subdivided into “social concepts”, “person knowledge,” and “emotion concepts.”

The following steps were carried out separately for the three analyses. First, to assess the incidence of unilateral and bilateral coordinates, each study was coded according to whether they reported (1) left ATL peaks only, (2) right ATL peaks only, or (3) bilateral ATL peaks. Chi-squared tests were then computed to test if the proportion of studies reporting a left ATL peak (i.e., unilateral left + bilateral) differed between the two types of study in each analysis (e.g., verbal vs. non-verbal input). This procedure was repeated for right ATL peaks.

### ALE Analysis

Analyses were performed with GingerALE 2.3 (available at http://brainmap.org/ale/ [date last accessed; 16 February 2015]; Laird et al. 2005; Eickhoff et al. 2009). This version uses a random-effects analysis to minimize potential biases caused by within-experiment or within-subject effects (Eickhoff et al. 2009). Spatial smoothing FWHM was determined based on the number of participants within each analysis and ranged from 8.66 to 12 mm (Eickhoff et al. 2009). Talariach coordinates were converted to the MNI space using the Tal2MNI (SPM) transform in GingerALE. Areas associated with significant ALE values were plotted on the high-resolution MNI152 template brain using MRICron (http://www.nitrc.org/projects/mricron [date last accessed; 16 February 2015]).

An overall activation likelihood map for all 97 studies was generated to show ATL coverage. This was thresholded using a false discovery rate (FDR) of *P* < 0.05 to correct for multiple comparisons. Then, to investigate shared versus specialized ATL function, three separate analyses were carried out (outlined above). In Analysis 1, studies were divided into two sets based on input modality, and the following analysis steps were performed:

1. Separate activation likelihood maps were generated for each set of studies (i.e., verbal and non-verbal) and were thresholded at *P* < 0.05 (FDR-corrected) to correct for multiple comparisons.
2. Subtraction analyses were conducted between these thresholded likelihood maps to investigate differences between verbal and non-verbal studies. Subtractions were thresholded at *P* < 0.05 (uncorrected) and were run using 5000 *P*-value permutations with a minimum cluster size of 200 mm. The conjunction image from this analyses represented regions which were equally likely to be activated by verbal and non-verbal studies.
3. Laterality analyses were also performed to identify regions within the ATL in which activation was more likely in the left hemisphere than the right and vice versa (Turkeltaub and Coslett 2010). To achieve this, the “*x*” coordinates for the verbal studies were left–right reversed (i.e., were multiplied by −1). A new subtraction analysis was then carried out, contrasting these “*x*-reversed” co-ordinates with the original likelihood peaks, to highlight regions for which activation for verbal inputs in the left hemisphere was more likely than activation of the homologous region in the right hemisphere (and vice versa). The conjunction image from this analysis represented regions which were equally likely to be active in both hemispheres. This process was repeated for the non-verbal studies. Cluster volume sizes from the laterality analyses for each dimension were extracted and plotted separately to illustrate the volume of tissue associated with bilateral versus lateralized activation.

For Analysis 2, the same steps were performed, except studies were divided based on whether they involved word retrieval or other processes. Finally, Analysis 3 repeated the same steps with studies divided according to semantic category (social vs. non-social).

## Results

Figure 1 shows the activation likelihood map for all 97 studies. Activation likelihood was greatest in the superior, lateral portions of the right and left ATLs, with low activation likelihood in the ventral ATLs. The ventral ATLs suffer from severe signal distortion and dropout in standard gradient-echo EPI fMRI and this, as well as other methodological factors, probably account for their under-representation here [see Visser, Jefferies, et al. (2010)]. One consequence of this inherent limitation in the literature is that studies using non-verbal pictorial stimuli, which are likely to rely heavily on the inferior aspects of the ATLs, may be under-represented. Recent studies that have used distortion-corrected fMRI to improve signal have demonstrated robust activation in ventral ATL regions for a range of semantic tasks (Binney et al. 2010; Visser and Lambon Ralph 2011; Visser et al. 2012). This aligns with evidence from PET studies (Devlin et al. 2000; Spitsyna et al. 2006) and with a recent study of hypometabolism in SD that linked the patient's semantic deficits with dysfunction centered on the anterior fusiform (Mion et al. 2010). It is likely that the paucity of activation observed in this area in the current meta-analysis reflects the reliance on (non-distortion-corrected) fMRI in the majority of studies, highlighting the need for future studies to take steps to improve imaging sensitivity in this region. For present purposes, however, this means that our analyses are necessarily focused on the lateral and superior aspects of the ATLs.

Next, the 97 studies were divided according to the predictions of the three specialized accounts of ATL function (“input modality,” “word retrieval,” and “semantic category”). To summarize what follows: (1) in each analysis, there was a high likelihood of bilateral ATL activation; and (2) variation within each analysis, reflecting the key feature of each specialized account, generated considerable common (bilateral) activation and relatively little specific activation. Beyond the overall bilateral distribution of activations, there was evidence of secondary specializations between and within the ATLs. Activation likelihood peaks for each ALE analysis are reported in Supplementary Table 2.

### Analysis 1: Modulation of ATL Function by Input Modality

**Figure 2.**
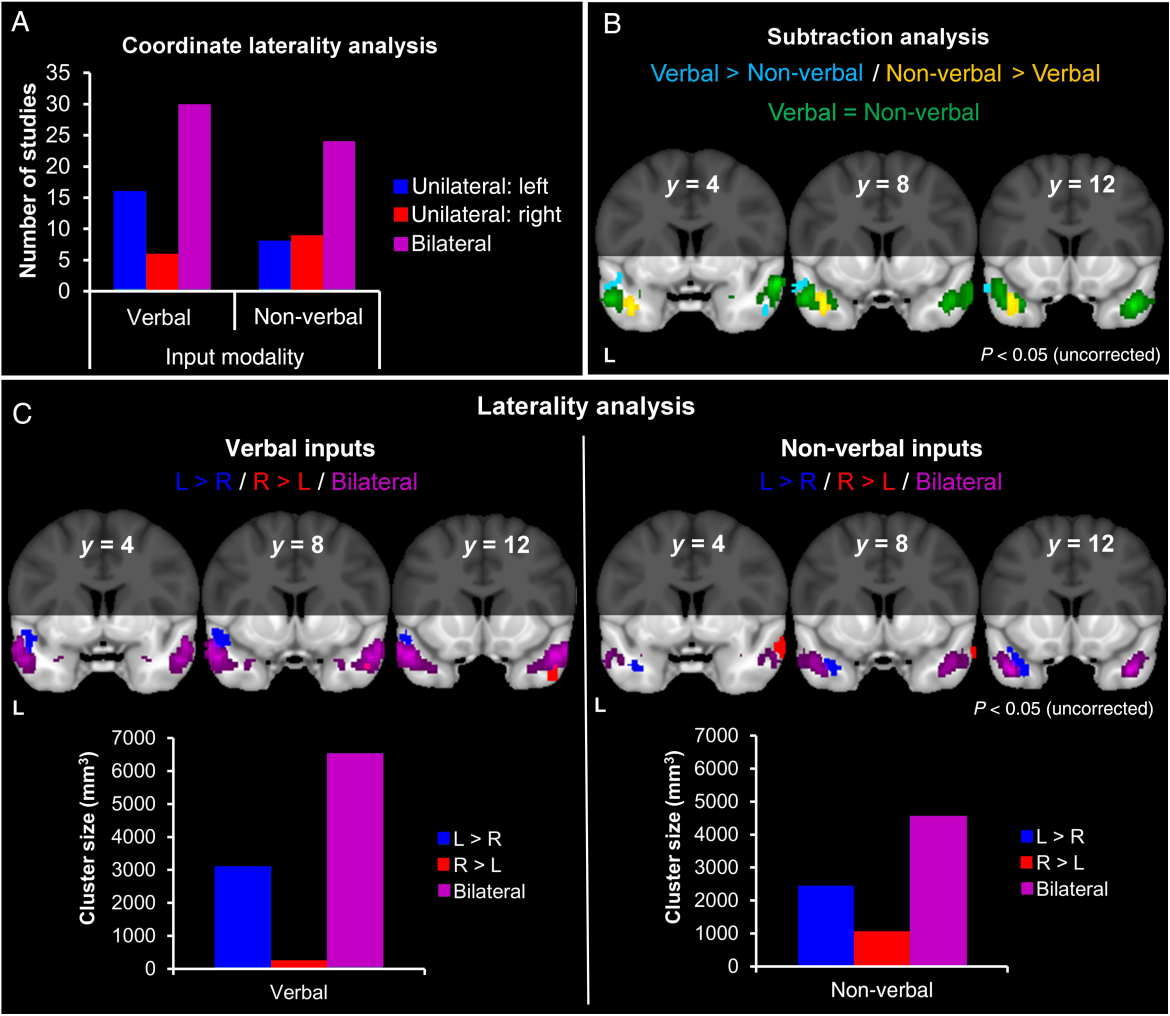
Influence of sensory input modality. (*A*) The number of studies contributing unilateral or bilateral coordinates. (*B*) Regions within the ATLs with greater activation likelihood for verbal inputs, compared with non-verbal inputs (light blue). Regions within the ATLs with greater activation likelihood for non-verbal inputs compared with verbal inputs (yellow). Regions with equal activation likelihood for both study types (green). (*C*) Laterality analysis for verbal and non-verbal inputs. Regions within the ATLs more likely to be active in the left, compared with the right, ATL (dark blue). Regions within the ATLs more likely to be active in the right, compared with the left, ATL (red). Regions within the ATLs with equal activation likelihood in both hemispheres (purple).

The bar chart in Figure 2*A* divides studies according to whether they reported unilateral left, unilateral right, or bilateral ATL peaks. The majority of studies across the input modalities reported bilateral peaks. Chi-squared analyses showed no significant differences between the proportion of “verbal” and “non-verbal input” studies reporting left ATL co-ordinates (*χ*^2^ = 1.84, *P* = 0.18) or in the proportion reporting right ATL coordinates (*χ*^2^ = 1.52, *P* = 0.22).

In the ALE analysis, both the “verbal input” and “non-verbal input” studies produced predominantly bilateral and highly overlapping activation, encompassing STG (BA 38) and MTG (BA 21; Supplementary Table 2).

Figure 2*B* shows the subtraction analysis, which reveals regions for which “verbal input” studies were more likely to produce activation than “non-verbal” (in light blue) and vice versa (yellow). The conjunction analysis (green) indicates areas with equal activation likelihood for both sets of studies. The majority of activated cortex, in both ATLs, was equally activated by both input modalities: This common area encompassed bilateral portions of STG (BA 38), extending along MTG (BA 21). Specialized regions for “verbal inputs” showed greater activation likelihood dorsally, along left STG (BA 38) and right MTG (BA 21), whereas specialized regions for “non-verbal inputs” showed greater activation likelihood more ventrally, focused in the left temporal pole (BA 38) and a smaller cluster in right parahippocampal gyrus (BA 34; Supplementary Table 3).

Figure 2*C* shows the laterality analyses for “verbal” and “non-verbal” inputs. The laterality analyses tested for regions in which there was greater activation likelihood in one hemisphere compared with the homologous region in the opposite hemisphere and, conversely, for those in which activation was equally likely in both hemispheres. The bar charts plot the volumes of clusters arising from these analyses. For both “verbal inputs” and “non-verbal inputs,” activation in the left and right was equally likely for large areas of superior, lateral ATLs (purple). Smaller, unilateral effects were observed across both sets of studies (Fig. 2*C*—red and dark blue and see also Table 2); however, the bar charts illustrate that these unilateral clusters were much smaller than the more prominent bilateral clusters. For “verbal inputs,” there was limited evidence for a small degree of left ATL specialization, as predicted by the input modality hypothesis (Gainotti 2012). For “non-verbal inputs,” the corresponding prediction of right ATL specialization was not supported.

**Table 2:** Activation likelihood clusters from the laterality analyses for each study type

Study type	Cluster no.	Cluster size (mm^3^)	Peak location (BA)	Peak MNI coordinates			*Z*-value
				*x*	*y*	*z*	
Input modality							
Verbal							
L > R	1	1224	STG (38)	−38	20	−18	2.46
			STG (38)	−32	20	−24	2.41
			STG (38)	−34	26	−28	1.97
			STG (38)	−52	20	−18	1.84
	2	1224	STG (22)	−49	6	−11	2.79
	3	664	Fusiform gyrus (20)	−38	−16	−32	2.41
R > L	1	264	ITG (20)	48	14	−38	2.07
			MTG (21)	50	10	−32	1.83
Bilateral	1	6548	STG (38)	±48	16	−28	0.03
			MTG (21)	±58	8	−20	0.03
			Parahippocampal gyrus (36)	±30	8	−30	0.02
			ITG (20)	±36	12	−34	0.02
Non-verbal							
L > R	1	2448	ITG (20)	−34	12	−29	3.24
			ITG (20)	−44	−2	−32	2.17
			MTG (21)	−52	0	−30	1.94
R > L	1	736	STG (38)	64	4	−20	2.73
	2	336	STG (38)	52	20	−30	2.35
Bilateral	1	4364	ITG (20)	±42	14	−34	0.03
			MTG (21)	±58	−4	−18	0.02
			MTG (21)	±56	4	−24	0.02
			MTG (21)	±58	4	−28	0.02
	2	200	Amygdala (28)	±26	0	−24	0.01
Word retrieval							
Word retrieval tasks							
L > R	1	2720	STG (22)	−46	6	−12	3.24
			STG (38)	−46	8	−18	2.99
			STG (38)	−48	4	−22	2.91
			MTG (21)	−52	0	−22	2.77
			MTG (21)	−56	−2	−16	2.75
R > L	1	1048	STG (38)	58	18	−28	2.73
Bilateral	1	260	MTG (21)	±48	8	−32	0.01
“Other” tasks							
L > R	1	1624	ITG (20)	−38	−8	−32	3.16
	2	1144	STG (38)	−34	14	−24	2.85
			STG (38)	−36	14	−18	2.82
R > L	1	1064	MTG (21)	64	0	−12	3.16
			MTG (21)	64	0	−16	3.09
Bilateral	1	6284	STG (38)	±42	14	−32	0.06
			MTG (21)	±52	8	−28	0.04
			MTG (21)	±56	6	−22	0.04
			MTG (21)	±58	−6	−18	0.03
			Amygdala (28)	±26	0	−24	0.02
			Parahippocampal gyrus (36)	±32	6	−28	0.02
			Hippocampus (28)	±20	−4	−18	0.01
Semantic category							
Non-social							
L > R	1	3240	STG (38)	−48	9	−11	3.54
			STG (38)	−44	22	−14	3.09
			STG (38)	−48	22	−14	2.95
			MTG (21)	−50	−4	−24	1.80
	2	1568	Fusiform gyrus (20)	−36	−10	−32	2.46
			Fusiform gyrus (20)	−32	−6	−34	2.22
			ITG (20)	−42	0	−30	2.17
			ITG (20)	−42	−20	−32	2.09
			Fusiform gyrus (20)	−32	0	−32	2.01
			ITG (20)	−44	−14	−28	1.91
Bilateral	1	5748	MTG (21)	±52	6	−30	0.03
			MTG (21)	±58	10	−20	0.03
			MTG (21)	±58	4	−18	0.03
			ITG (20)	±36	12	−32	0.02
Social							
R > L	1	1176	MTG (21)	65	3	−21	3.54
L > R	1	1088	STG (38)	−36	14	−26	3.54
Bilateral	1	6708	STG (38)	±46	18	−26	0.03
			ITG (20)	±46	10	−30	0.03
			MTG (21)	±54	8	−28	0.02
			MTG (21)	±58	−4	−18	0.02
	2	748	Amygdala (28)	±26	0	−24	0.02
			Parahippocampal gyrus (36)	±34	4	−24	0.02
			Amygdala (28)	±20	−4	−18	0.01

Note: Clusters are marked as having greater activation likelihood in the left hemisphere compared with the homologous region in the right hemisphere (L > R) and vice versa (R > L) and regions more likely to be active in both hemispheres.

Finally, the sensory input subanalysis, in which verbal studies were subdivided into written versus auditory words, revealed some differences between these two stimulus classes. While both written and spoken words showed bilateral activation across STG (BA 38) and MTG (BA 21; Supplementary Fig. 1*A* and Table 4), there was a tendency for this activation to be more focused on the left ATL in the case of written words.

### Analysis 2: Modulation of ATL Function by Word Retrieval

**Figure 3.**
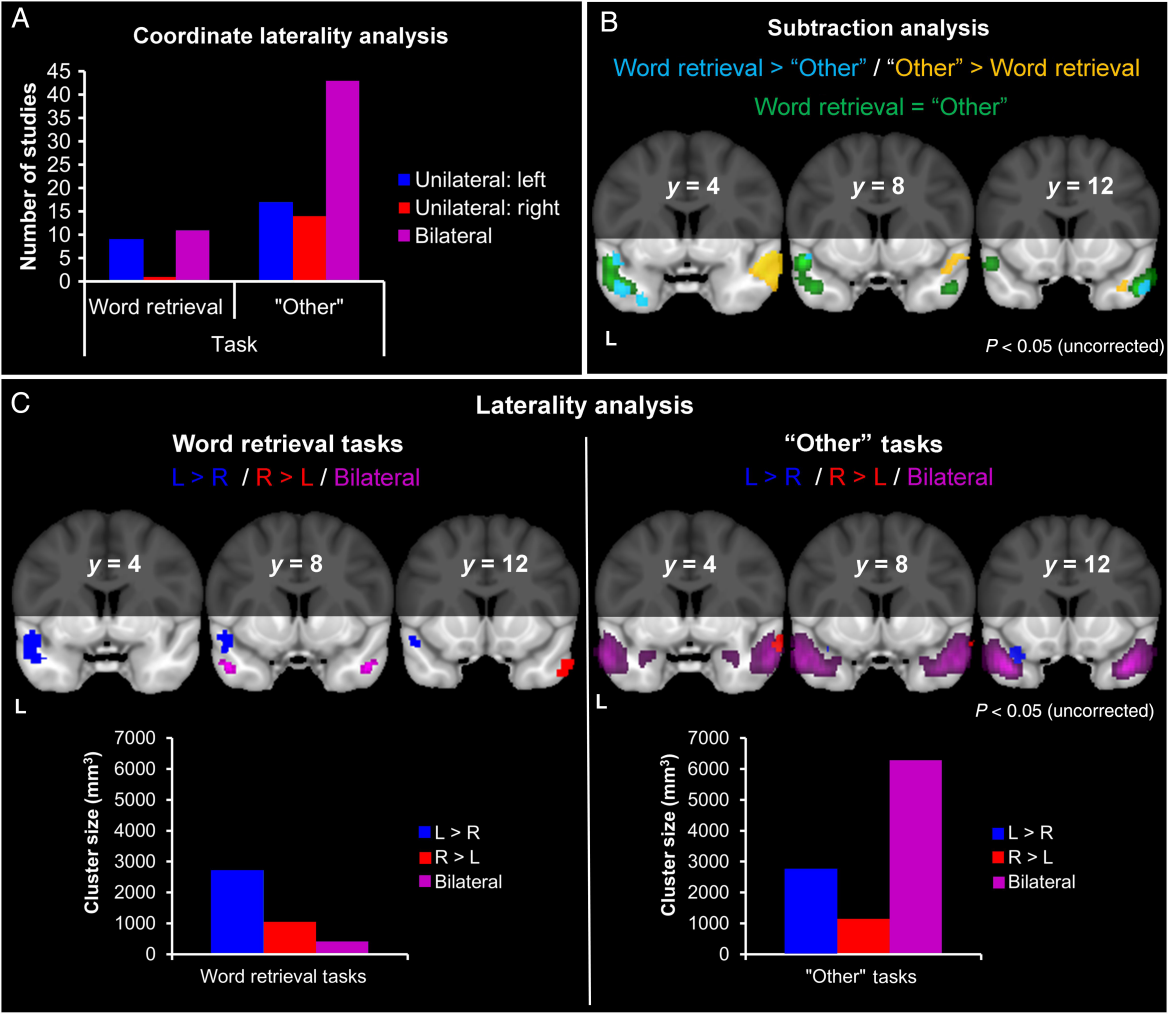
Influence of word retrieval. (*A*) The number of studies contributing unilateral or bilateral coordinates. (*B*) Regions within the ATLs with greater activation likelihood for word retrieval, compared with other tasks (light blue). Regions within the ATLs with greater activation likelihood for other tasks compared with word retrieval (yellow). Regions with equal activation likelihood for both study types (green). (*C*) Laterality analysis for word retrieval and other tasks. Regions within the ATLs more likely to be active in the left, compared with the right, ATL (dark blue). Regions within the ATLs more likely to be active in the right, compared with the left, ATL (red). Regions within the ATLs with equal activation likelihood in both hemispheres (purple).

The bar chart (Fig. 3*A*) indicates within “word retrieval” studies, there were similar numbers of bilateral and left hemisphere-only studies. In contrast, the majority of “other” studies reported bilateral peaks. Chi-squared analyses showed no significant differences between the proportion of “word retrieval” and “other” studies reporting left ATL co-ordinates (*χ*^2^ = 2.47, *P* = 0.12), or right ATL coordinates (*χ*^2^ = 3.25, *P* = 0.07).

In the ALE analysis, both the “word retrieval” and “other” studies produced predominantly bilateral and highly overlapping activation, encompassing STG (BA 38) and MTG (BA 21; Supplementary Table 2).

Figure 3*B* shows the subtraction between “word retrieval” and “other” tasks. Equal activation likelihood for both study types was shown around left STG (BA 38) and, to a lesser extent, bilateral MTG (BA 21). Specialized regions for “word retrieval” showed greater activation likelihood along left STG (BA 38), extending into the left temporal pole and right MTG (BA 21), whereas those for “other” tasks showed greater activation likelihood along right MTG (BA 21), extending toward the temporal pole bilaterally (Supplementary Table 3).

Figure 3*C* shows the laterality analyses for “word retrieval” and “other” tasks. For “word retrieval,” left and right activation was equally likely in some areas of STG (BA 38) and MTG (BA 21); however, these bilateral regions were outweighed by a large area of STG that showed increased activation likelihood in the left ATL compared with the right ATL, and a smaller, more anterior STG region that showed the opposite effect (Table 2). In all, the volume of the left ATL cluster far exceeded that of the right ATL or the bilateral clusters, indicating that tasks involving word retrieval activate large areas of the left ATL, to a greater degree than the right. This provides support for the notion that word retrieval tasks rely heavily on the left ATL (Lambon Ralph et al. 2001; Damasio et al. 2004). For “other” tasks, the majority of cortex was equally likely to be active in the left and right ATLs (purple), with smaller unilateral effects observed in both hemispheres (Table 2). The bar chart illustrates that these unilateral clusters were outweighed by the prominent bilateral cluster.

The secondary analysis subdivided the “word retrieval” studies into reading versus naming studies and showed that reading studies principally activated the left ATL, whereas naming studies produced more bilateral activation (Supplementary Fig. 1*B* and Table 4). Therefore, as we also observed in the previous analysis, there was a tendency for processing of written words to produce more left-lateralized activation.

### Analysis 3: Modulation of ATL Function by Semantic Category

**Figure 4.**
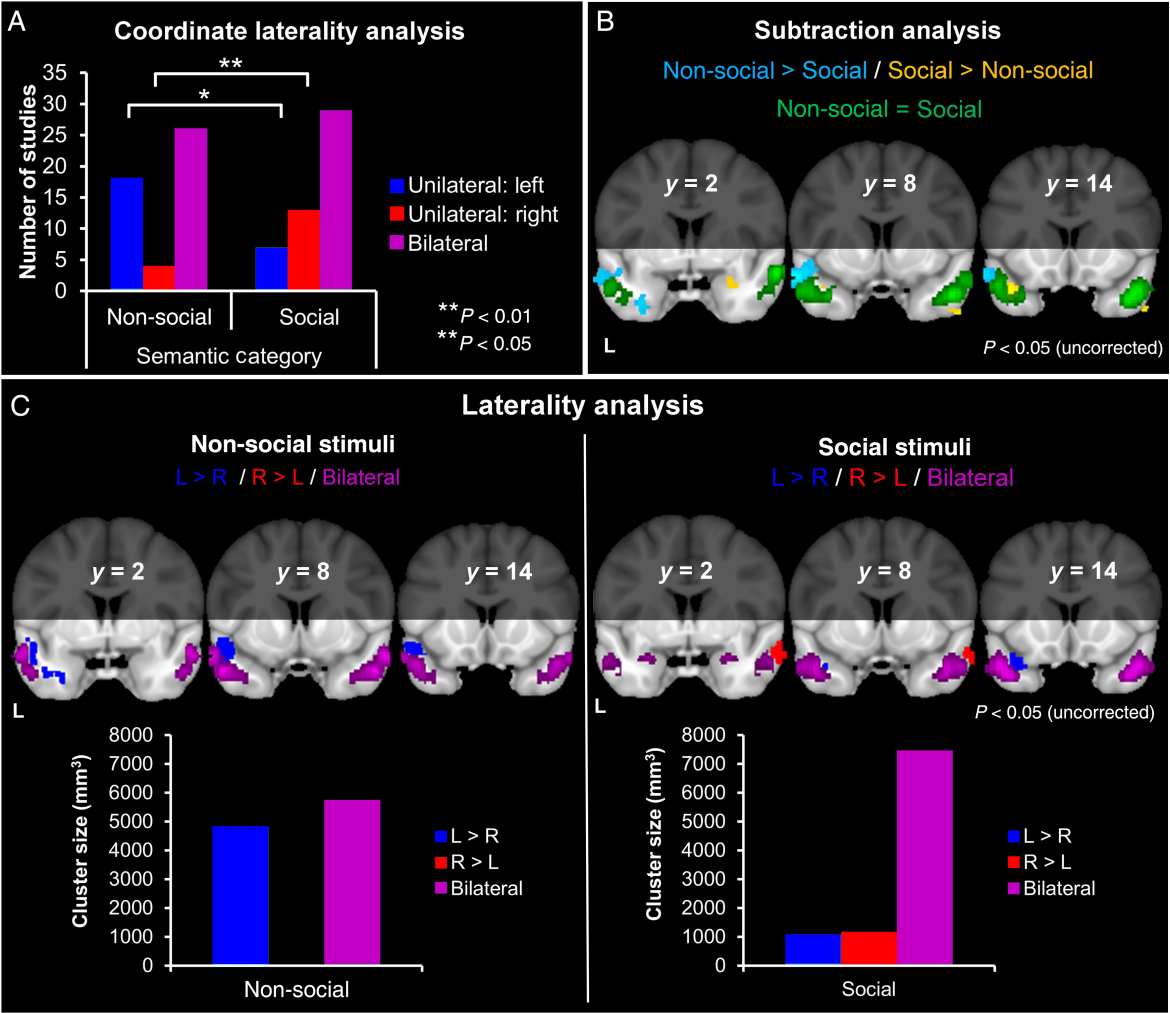
Influence of semantic category. (*A*) The number of studies contributing unilateral or bilateral coordinates. (*B*) Regions within the ATLs with greater activation likelihood for non-social semantics, compared with social semantics (light blue). Regions within the ATLs with greater activation likelihood for social semantics compared with non-social semantics (yellow). Regions with equal activation likelihood for both study types (green). (*C*) Laterality analysis for non-social and social semantics. Regions within the ATLs more likely to be active in the left, compared with the right, ATL (dark blue). Regions within the ATLs more likely to be active in the right, compared with the left, ATL (red). Regions within the ATLs with equal activation likelihood in both hemispheres (purple).

The bar chart (Fig. 4*A*) indicates that the majority of studies across semantic categories reported bilateral peaks. There was, however, some asymmetry among studies that reported unilateral peaks, with non-social studies tending to report left rather than right peaks and social studies tending to report right rather than left peaks. Chi-squared analyses showed that “non-social semantic” studies were indeed more likely to elicit left ATL peaks than “social semantic” studies (*χ*^2^ = 5.55, *P* = 0.02), whereas “social semantic” studies were more likely to produce right ATL coordinates than “non-social semantics” (*χ*^2^ = 6.83, *P* = 0.01).

In the ALE analysis, both the “non-social semantic” and “social semantic” studies produced predominantly bilateral and highly overlapping activation, encompassing STG (BA 38) and MTG (BA 21; Supplementary Table 2).

Figure 4*B* shows the subtraction between “non-social” and “social semantics.” Equal activation likelihood for both study types encompassed large parts of bilateral MTG (BA 21) and STG, extending into the temporopolar cortex. Specialized regions for “non-social semantics” showed greater activation likelihood in the left STG (BA 38) and a smaller part of ITG (BA 20). In contrast, specialized regions for “social semantics” showed greater activation likelihood in more polar and more medial areas, encompassing left STG (BA 38), bilateral hippocampus, and right ITG (BA 20; Supplementary Table 3).

Figure 4*C* shows the laterality analyses for “non-social semantics” and “social semantics.” For both the “non-social and “social semantic” studies, bilateral ATL activations (purple) were most prominent. Again, smaller unilateral effects were observed in the right and left ATLs for both study types (Table 2). For the non-social studies, there was a combination of bilateral clusters and a left (superior) ATL cluster, suggesting a degree of leftward bias in an otherwise bilateral picture. For social studies, the bulk of the ATL tissue showed equal activation likelihood in left and right. There was, therefore, limited support for the idea that social concepts preferentially activate the right ATL (Olson et al. 2007).

Across all three social subcategories (“social concepts,” “person knowledge,” and “emotion concepts”), activation likelihood was bilateral and overlapping, encompassing portions of the STG (BA 38) and MTG (BA 21; Supplementary Fig. 1*C* and Table 4).

### Laterality Outside the ATLs

**Figure 5.**
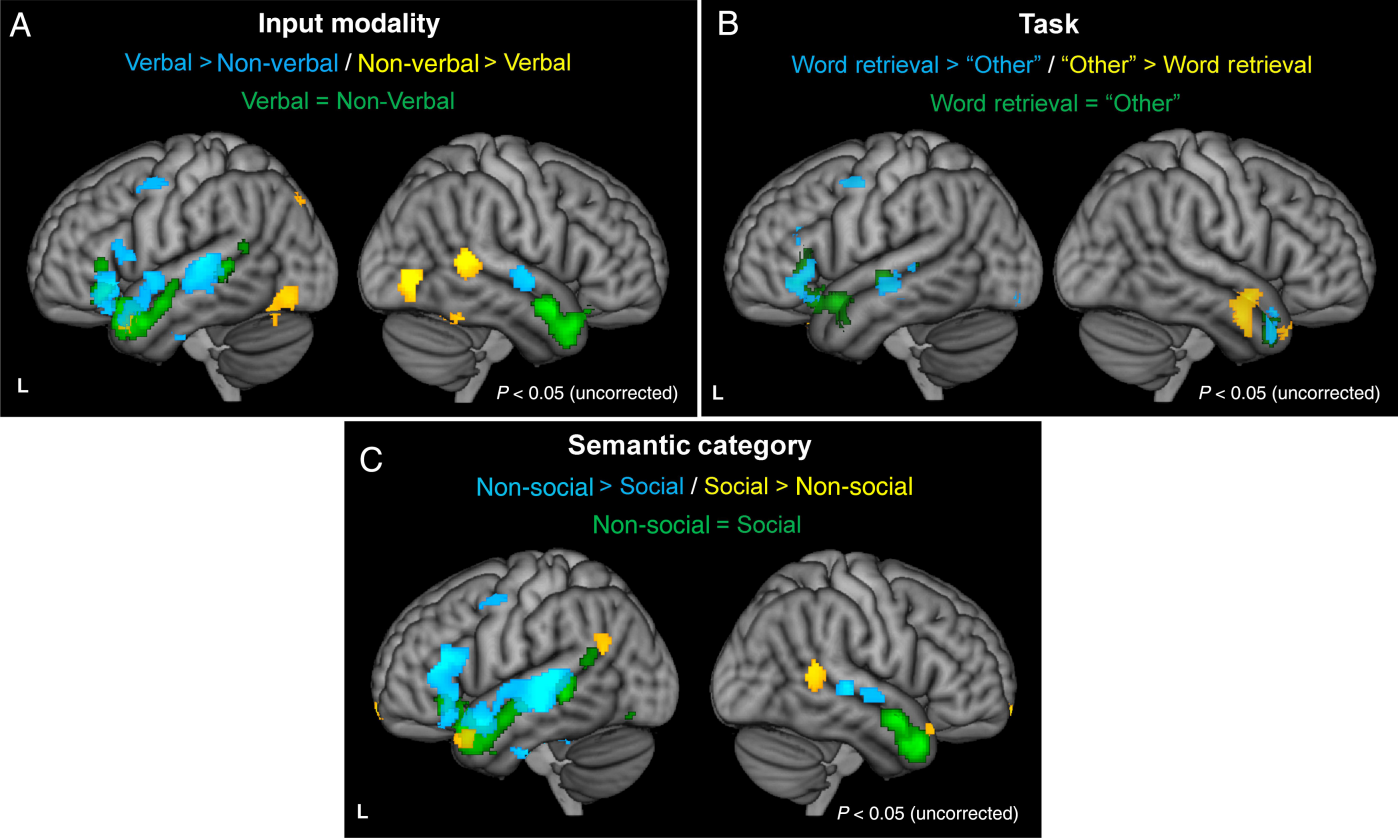
Subtraction analyses across the whole brain. (*A*) “Input modality”: Regions with greater activation likelihood for verbal inputs, compared with non-verbal (blue). Regions with greater activation likelihood for non-verbal inputs compared with verbal (yellow). (*B*) “Word retrieval”: Regions with greater activation likelihood for word retrieval, compared with other tasks (blue). Regions with greater activation likelihood for other tasks compared with word retrieval (yellow). (*C*) “Semantic category”: Regions with greater activation likelihood for non-social semantics, compared with social (blue). Regions with greater activation likelihood for social semantics compared with non-social (yellow). In all panels, regions with equal activation likelihood for both study types (green).

This analysis investigated effects of each study type across the whole brain to contrast against the previous ATL-focused analyses (Fig. 5). Considering input modality first, there was specialization for “verbal inputs” in areas of the left temporal and prefrontal cortex, predominantly in the left hemisphere. In contrast, “non-verbal inputs” (dominated by picture-based studies) were more likely to activate the posterior ventral temporal lobe bilaterally, although this was stronger in the right hemisphere. Specialized regions for “word retrieval” were found almost exclusively in the left hemisphere along the length of the STG extending into the inferior frontal gyrus, whereas “other” tasks showed greater activation likelihood in right ATL regions and in the orbitofrontal cortex. Greater activation likelihood for “non-social semantics,” relative to social semantics, was observed in large areas of temporal and prefrontal cortex, predominantly in the left hemisphere. Specialization for “social semantics” was evident in the superior temporal poles and temporoparietal junction bilaterally. In summary, in contrast to other parts of the brain, the ATLs tended not to show hemispheric specialization along any of the three specialized accounts of ATL function, reinforcing the idea that the two ATLs operate as an integrated, bilateral system.

In addition, the whole-brain analysis revealed that studies of every type reliably activated the inferior prefrontal cortex (in particular, pars orbitalis) and the posterior MTG, suggesting that these areas are also involved in semantic cognition. A detailed consideration of the function of these areas is beyond the scope of the present study; however, both have been linked to top-down executive influences on the retrieval and manipulation of semantic knowledge (Thompson-Schill et al. 1997; Badre and Wagner 2002; Jefferies and Lambon Ralph 2006; Jefferies 2013).

## Discussion

This study tested four accounts of the roles of right and left ATLs in supporting conceptual knowledge. While some researchers hold that the ATLs form an integrated bilateral system for representing knowledge (Patterson et al. 2007; Schapiro et al. 2013; Lambon Ralph 2014), others have proposed a degree of hemispheric specialization, organized by input modality, word retrieval, or semantic category (Damasio et al. 2004; Olson et al. 2007; Gainotti 2012). We tested these predictions in an ALE meta-analysis of 97 neuroimaging studies. The most striking finding was that, predominately, bilateral overlapping activations were observed across all three proposed dimensions of specialization. Secondary to this bilateral pattern, there were two more subtle graded hemispheric specializations: (1) A left hemispheric bias for tasks requiring word generation and (2) some evidence that written word input is more likely to activate the left ATL. In contrast, there was no evidence of hemispheric specialization for social versus non-social concepts. Although extending further into anteromedial ATL regions, activation likelihood for social concepts was firmly bilateral.

**Figure 6.**
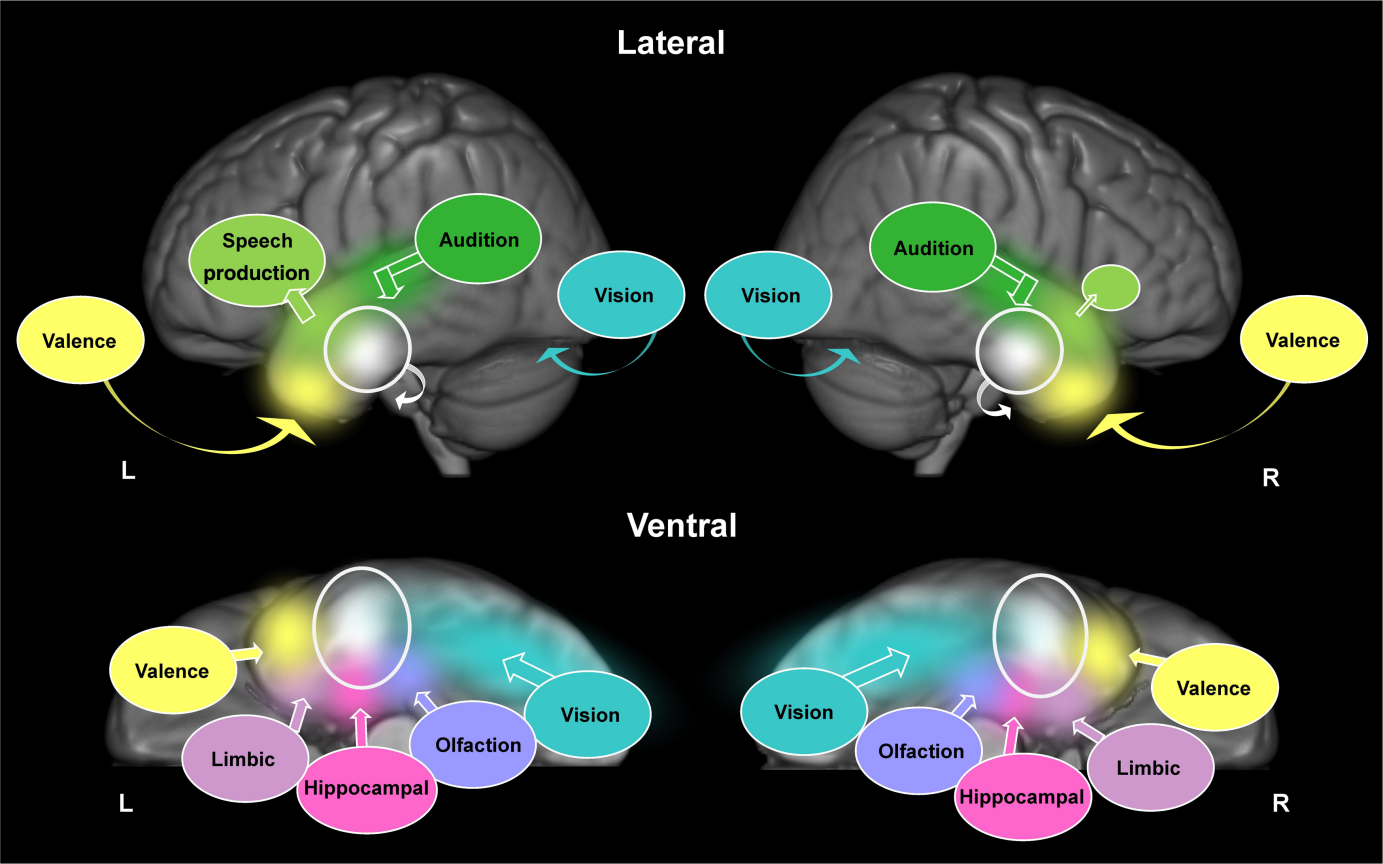
Illustration of the bilateral, yet graded representation of conceptual knowledge across both ATLs; shown on lateral (top) and ventral (bottom) views. The ventrolateral portions of the ATLs, bilaterally (white circles), receive converging inputs from primary sensory cortices and medial temporal structures (colored circles). The different colors represent information from these different input regions converging upon the ventrolateral ATLs; eventually becoming mixed (white). Bold arrows illustrate the direction of convergence. Curved arrows illustrate the direction of activation that cannot be seen on the lateral surface, for example, visual information travels along the ventral surface of the temporal lobes via the fusiform gyrus. Differential connectivity is illustrated as speech output regions in the frontal lobes being larger in the left hemisphere, compared with the right hemisphere (light green circles). For simplicity, only inputs relevant to the current meta-analysis are illustrated; connections to other important nodes in the semantic system, including regions involved in cognitive control, have been omitted.

None of the existing accounts of ATL function can fully account for these results. At face value (though see the “Caveats” subsection below), the very clear bilateral activation likelihood maps found in this study seem to be inconsistent with notions of strong specialized distinctions between the left and right ATLs, as would be expected on an extreme version of some theories (Tranel et al. 1997; Gainotti 2007; Olson et al. 2007). Likewise, the presence of second-order graded specialization refutes an extreme, undifferentiated bilateral account in which there are no differences between the left and right ATLs, whatsoever. Instead, we argue that the results fit best with a neurocomputational framework for ATL function (summarized in Fig. 6), which incorporates a basic principle of bilateral representation, but allows for some graded functional specialization emerging as a result of asymmetric connectivity between ATL subregions and primary input/output areas (Lambon Ralph et al. 2001; Plaut 2002; Visser and Lambon Ralph 2011; Binney et al. 2012; Visser et al. 2012; Pascual et al. 2015; Schapiro et al. 2013; Lambon Ralph 2014).

### Conceptual Knowledge: Why Two Transmodal ATLs Are Better Than One

The main result from the current study is that conceptual knowledge of various types activates an ATL system that is both bilateral and transmodal. The role and potential importance of a transmodal representational hub has been discussed and computationally implemented in previous studies (Rogers et al. 2004; Patterson et al. 2007; Lambon Ralph, Sage, et al. 2010; Lambon Ralph 2014). In short, a transmodal hub supports the complex, nonlinear computations required to integrate multiple sources of verbal and non-verbal information into generalizable coherent concepts. Why is a bilateral hub beneficial for conceptual representation? One potential advantage of such a system is that it is more resistant to damage and, indeed, both human and primate data indicate that unilateral ATL damage/resection has much less of an effect on semantic performance than bilateral damage (Brown and Schafer 1888; Kluver and Bucy 1939, 1937; Terzian and Ore 1955; Lambon Ralph, Cipolotti, et al. 2010; Lambon Ralph et al. 2012). While the consequences of unilateral damage are certainly not trivial, bilateral damage is typically necessary to produce the profound deficits seen in, for example, SD. Using a neurocomputational dual hub-and-spoke model of semantic representation, Schapiro et al. (2013) provided a computational demonstration and explanation for this advantage by showing that bilateral lesions produced greater semantic impairments than unilateral lesions, even when the total amount of damage was controlled for. During learning, this model was able to use two transmodal “demi-hubs”—analogous to left and right ATLs—to mediate between knowledge of objects coded in different sensory modalities. Following unilateral damage to one “demi-hub”, semantic performance was supported by high fidelity and strong propagation of activation between the undamaged “demi-hub” and the various input/output units. Consequently, this strong and accurate activation propagation compensated, at least in part, for the weakened and distorted activations within the damaged demi-hub. In contrast, bilateral damage to both demi-hubs created representational distortion and activation weakness across the entire semantic system, resulting in much worse performance. Although explored in the context of semantic representation, the advantages for having a bilateral, partially redundant system have also been highlighted in other cognitive domains such as episodic memory (Scoville and Milner 1957) and visual recognition (Plaut and Behrmann 2011; Behrmann and Plaut 2013)—suggesting that this may be a more general neurocomputational principle (Schapiro et al. 2013).

One remaining question related to a bilateral conceptual knowledge system is why some studies report ATL effects limited to one hemisphere, or report highly specific deficits following unilateral damage. In the context of neuropsychology, a small number of case studies have reported deficits following unilateral lesions which are specific to one modality (Ellis et al. 1989; Mesulam et al. 2013). While these studies appear to provide support for a more lateralized view of ATL function, one explanation for the apparent sparing of other aspects of knowledge may be that the methods of testing were not sensitive enough. The importance of maximizing testing sensitivity, particularly in patients with unilateral damage, has been highlighted in two recent studies (Behrmann and Plaut 2014; Lambon Ralph et al. 2012). Lambon Ralph et al. (2012) found that patients with unilateral ATL resection show subtle transmodal semantic deficits, which were generally observed as selective slowing of reaction times and only resulted in increased error rates in particularly demanding semantic tasks (e.g., understanding low frequency and abstract words). Similarly, a number of neuroimaging studies included in this meta-analysis only reported peaks in one ATL (e.g., Devlin et al. 2000; Damasio et al. 2001; Vandenberghe et al. 2002; Elfgren et al. 2006). If the semantic system is truly bilateral, why do some semantic studies observe unilateral activation? Setting aside the influence of using written words as stimuli or requiring word retrieval (see below), a potential answer is that any individual study is susceptible to false positives and false negatives. While false positives can be rigorously controlled through good statistical practice, false negatives are more difficult to avoid and become more of a danger when stringent whole-brain corrections are performed. In the minority of studies that reported unilateral ATL activation, it is entirely possible that subthreshold activation was present in the opposite ATL. Indeed, our meta-analysis suggests that it is unwise to conclude that a particular task activates one ATL and not the other, on the basis of a single study, unless activation levels in each ATL are compared with one another statistically. The experience from reviewing all of the studies included in this meta-analysis suggests that such tests are rarely performed [but see Tsukiura et al. (2010) for an exception].

### Secondary Specializations Between the ATLs

Beyond the overall bilateral transmodal pattern of ATL activation, we observed clear evidence for two graded functional specializations between the ATLs: Studies presenting written words or requiring word retrieval were more likely to activate the left ATL than the right. These results, particularly those in relation to the left ATL and word retrieval, align closely with repeated observations of greater word-finding difficulties in patients with left greater than right ATL damage (e.g., left > right in neurodegenerative bilateral diseases or in comparisons of left versus right ATL resection cases: Lambon Ralph et al. 2001; Seidenberg et al. 2002; Lambon Ralph, Cipolotti, et al. 2010; Drane et al. 2013; Lambon Ralph et al. 2012). Likewise, the left bias for written word processing found in this meta-analysis reproduces the results of spoken (bilateral) versus written (left) word processing found in a previous MEG study (Marinkovic et al. 2003). This latter result is important, methodologically, given that neuroimaging studies of semantic processing commonly use written word stimuli. This is perhaps unsurprising given that written words allow a full range of concepts to be probed and are trivially easy to source when constructing experiments but, in doing so, the resultant neuroimaging data may be artificially left-biased.

We propose that these hemispheric specializations could arise in a bilateral semantic system as a consequence of asymmetric connectivity [for implemented computational models of this proposal, see Lambon Ralph et al. (2001) and Schapiro et al. (2013)]. These differences in connectivity could follow from two potentially linked sources. The first is asymmetry in the key white matter pathways. Both the ventral language pathway (tracts coursing through the extreme capsule complex) and the dorsal language pathway (arcuate fasciculus) are asymmetrically biased toward the left hemisphere (Parker et al. 2005; Catani et al. 2007). The second source would arise from left-lateralized neural systems for speech production (Blank et al. 2002), leading to greater functional connectivity between them and the left ATL. Even if the white matter structural connectivity was equal across left and right hemispheres, functional connectivity would still become left ATL-biased if the speech output system is predominantly rooted in left hemisphere structures. It is likely that these same neurocomputational principles can be extended to explain the left ATL bias for written word semantic tasks, as well (Plaut and Behrmann 2011). It is already established that there are graded left–right differences in posterior ventral occipito-temporal (vOT) for different categories of visual stimuli, including a left bias for written words (Cohen et al. 2002). Given that the vOT provides the visual input to a transmodal ATL hub, it is likely that any asymmetries in the input will have a corollary effect on ATL activation, making it left-biased. The source of the left vOT bias for written word processing is debated. It seems to emerge during the process of learning to read (such that face recognition, for example, then becomes more right lateralized as a consequence: Dundas et al. 2013). There is an intriguing possibility, therefore, that the left bias for written words also relates directly to speech production; in learning to read, children are initially taught to read aloud (i.e., “speak the words”). Thus, as per the biases in connectivity for (semantically driven) word retrieval, written word recognition and reading aloud might also become left biased.

In contrast to word retrieval and written word processing, we found no support for a corresponding right ATL bias for either non-verbal information (Gainotti et al. 2003; Damasio et al. 2004) or socially related concepts (Olson et al. 2007, 2013); instead both were distinctly bilateral. Again, this might reflect the symmetry of the relevant connections from visual association cortex into the left and right ATL. Thus, for example, the inferior longitudinal fasiculus (the principal white matter bundle connectivity occipital and ATL regions) shows no hemispheric asymmetry (Schmahmann et al. 2007; Thiebaut de Schotten et al. 2011). Accordingly, visual perceptual information may be better thought of as being graded along the posterior–anterior axis, with greater specialization in the posterior temporal cortex because of increased proximity to the primary visual cortex (Mesulam 1998; Plaut 2002). For the formation of social and emotional concepts, inputs from frontal and limbic regions—primarily delivered through the uncinate fasciculus—will be important (Moran et al. 1987; Binney et al. 2012; Von Der Heide Skipper, Klobusicky, et al. 2013). The laterality of the uncinate fasciculus is currently unclear as, although one postmortem study found this tract to be larger in the right than left hemisphere (Highley et al. 2002), recent diffusion-weighted imaging studies have failed to replicate this asymmetry, convincingly (Kubicki et al. 2002; Hasan et al. 2009).

Finally with regard to left–right ATL differences, we should note that there does appear to be one disparity between the functional neuroimaging results (as analyzed here) and neuropsychology literature. As described above, the two literatures align very clearly with regard to the leftward bias for speech production/confrontational naming. Given that both L > R and R > L SD patients are reported to have altered social behavior and neuropsychiatric features when formally tested (Chan et al. 2009), the bilateral activations for social concepts, found in this meta-analysis, also seem to be consistent with the neuropsychological literature. In contrast, the lack of a rightward activation bias for face-based stimuli is potentially surprising given reports of face recognition deficits in patients with chronic or progressive damage to the right ATL (Ellis et al. 1989; Evans et al. 1995; Tranel et al. 1997; Damasio et al. 2004). A recent study directly compared left and right ATL-resected temporal lobe epilepsy patients on various aspects of face processing (Drane et al. 2013). In keeping with the current meta-analysis and other findings (see above), the left ATL-resected patients were much more anomic than their right ATL counterparts. The reverse was also true, however, for face familiarity and identification; although the left ATL cases were mildly impaired when compared with controls, the right ATL group was significantly worse. Future targeted research is needed to explore this puzzle in more detail. As noted below, bilateral activation might be expected if different components of a task (e.g., face recognition vs. name retrieval) were supported separately by each hemisphere. An alternative hypothesis arises from considering some details in the Drane et al. (2013) results, which may prove critically important. Briefly, as described above, following the convergent inputs and outputs of the ventrolateral ATL region, the resulting representations will tend to be transmodal in nature (Binney et al. 2012; Lambon Ralph 2014). The face recognition deficits of the right ATL cases in the Drane et al. (2013) study, however, were not transmodal in form. Instead, the patients demonstrated a classical prosopagnosic impairment in which identification of familiar people was impaired when presented (visually) as a face but was much better when the same information was probed from a different modality (e.g., the spoken name). This suggests some kind of disruption between visual input and the semantic system rather than a deficit within the semantic system itself. Following the impact that differential neural/functional connectivity can have on performance (see above), it may be possible that the right ATL patients demonstrated poor visual face recognition because the visual input (from vOT regions) is right biased even if person knowledge itself is bilaterally distributed across the ATLs (the latter would be consistent with the fact that multiple modalities contribute to our knowledge of familiar people). If this hypothesis is correct, then the right ATL patients' prosopagnosia would follow from the same neurocomputational principle of differential connectivity which we and others have used to explain the left ATL bias for speech production (Lambon Ralph et al. 2001; Schapiro et al. 2013). A caveat to this discussion is that Drane et al. (2013) did not test person recognition from any other modalities (e.g., voices). It is therefore not clear that the right ATL patients had a purely prosopagnosic impairment. Indeed, a review recent has shown that, in the cases where faces and voices are tested together, right ATL patients often exhibit a transmodal recognition deficit, rather than a purely visual deficit affecting face recognition (Gainotti 2013).

### Secondary Specializations Within the ATLs

We also found graded specializations *within* the ATLs and again, these gradations were subtle and secondary to the overall picture of bilateral and transmodal activations. Specifically, activation extended dorsally toward STG when inputs were verbal and extended ventrally toward ITG when inputs were non-verbal (primarily pictures). This finding is consistent with the results of recent individual fMRI studies (Skipper et al. 2011; Visser and Lambon Ralph 2011; Visser et al. 2012) and a previous meta-analysis (Visser, Jefferies, et al. 2010). In addition, activation extended further into the temporopolar cortex when stimuli had social connotations. Like inter-ATL differences, we propose that these intra-ATL gradations arise from differential connectivity with primary sensory and limbic regions (Fig. 6). The ATLs are richly connected to widely distributed structures, including primary sensory regions in posterior temporal and occipital lobes, medial structures (limbic system, olfactory cortex, and episodic systems), and frontal systems, implicated in social behavior, valence, executive function, and various aspects of language processing (Gloor 1997; Ding et al. 2009; Blaizot et al. 2010; Binney et al. 2012; Pascual et al. 2015). Variations in the degree to which particular ATL subregions are connected to each of these areas could account for these patterns of graded specialization [see Plaut (2002); Visser and Lambon Ralph (2011); Binney et al. (2012); Visser et al. (2012)]. Although not applied to the ATL region per se, Plaut's (2002) computational model of semantic processing demonstrated the principle that, while an entire representational region may take part in semantic computations, subregions will become differentially important for divergent subsets of semantic activities depending on their distance/strength of connection to the critical input/output cortical areas.

Figure 6 attempts to integrate some of the known long-range connectivity patterns to the ATL and to sketch the resultant graded representational hub centered on the ventrolateral ATL bilaterally [see Binney et al. (2012) for a more detailed consideration of this idea and the intra- and inter-ATL connectivity]. On this view, the dorsal ATL's partial specialization for auditory-verbal stimuli emanates from stronger connections to the auditory association cortex in posterior STG and speech production areas in the inferior frontal gyrus (via the middle longitudinal fasciculus; see Fig. 6, green). Conversely, greater specialization for visual stimuli in more ventral areas would arise from inferior longitudinal fasiculus connections between the occipital and occipito-temporal regions (Fig. 6, blue). Likewise, the extension of the ALE maps for social concepts into anteromedial temporopolar regions would fit with stronger connections (via the uncinate fasiculus; Fig. 6, yellow) to the more medial (limbic; Fig. 6, yellow/purple) temporal lobe regions as well as orbitofrontal and insular areas [for studies of ATL functional and structural connectivity in human and non-human primates, see Moran et al. (1987); Binney et al. (2012); Pascual et al. (2015)]. This latter proposal resonates closely with previous suggestions from fMRI that temporopolar regions are crucial in the representation of social and emotionally laden abstract concepts due to strong connectivity with limbic and orbitofrontal regions (Moll et al. 2005; Zahn et al. 2007; Vigliocco et al. 2014).

### Caveats

In this study, we have focused on data arising from functional imaging studies in healthy individuals, rather than the effects of neurological damage. The principal advantages of functional neuroimaging are (1) they provide data about the organization of the unimpaired cognitive system, whereas brain damage can induce plasticity and compensatory processes that result in functional reorganization in patients (Duffau 2005; Keidel et al. 2010) and (2) they provide coverage of the whole brain (though see next paragraph for some limits to this), whereas lesion studies only provide data on the areas that happen to be damaged in a particular patient cohort. Conversely, the chief advantage of lesion studies is that they can demonstrate the *necessity* of a brain area for a particular function, whereas activations are correlational in nature. The tasks used in both types of study are also typically composed of multiple cognitive processes, which might draw on different neural resources. This point is particularly important when we consider the word retrieval tasks investigated as part of this study. Naming a picture is a complex process that involves considerable semantic processing in identifying the object as well as in retrieving its name. Therefore, even if there was a strongly specialized system (in which the left ATL was only involved in name retrieval and the right only in identifying the object), the multiple cognitive components that underpin this task could give rise to bilateral activation. Individual studies can be designed to tease apart the contributions of brain regions to these different processes (by, for example, devising a control task thought to require the same cognitive processes with the exception of the name retrieval) and/or might require MEG or electrophysiological techniques with better temporal resolution than fMRI. This level of experimental control is impossible, however, in a large-scale meta-analysis that includes data from a diverse set of studies that have employed very different paradigms and stimuli. Conversely, the great advantage of meta-analyses is that they allow hypotheses to be tested on a much larger dataset than could be collected in a single study, thus reducing the probability of false positives or false negatives.

It is important therefore to be mindful of the advantages and limitations of each technique and to seek convergence in the conclusions offered by each. The present results offer a good deal of convergence. As discussed earlier, the pattern of overall bilateral recruitment of the ATLs across different stimulus types is consistent with the notion of a bilateral distributed system, which was developed based on observations of the consequences of unilateral versus bilateral ATL damage (Lambon Ralph, Cipolotti, et al. 2010; Schapiro et al. 2013). Similarly, the left ATL activation bias for word retrieval tasks is consistent with findings in various patient groups, including the consequences of left-dominant ATL atrophy in SD (Lambon Ralph et al. 2001) and unilateral resection in patients with temporal lobe epilepsy (Seidenberg et al. 2002; Drane et al. 2013; Lambon Ralph et al. 2012). There do remain some areas of apparent disagreement between the results of this neuroimaging meta-analysis and the consequences of ATL damage. Most notably, many clinical studies have reported that abnormalities in social behavior are more pronounced in patients with greater right ATL damage (Edwards-Lee et al. 1997; Miller et al. 1997; Chan et al. 2009; Zahn et al. 2009). In contrast, our meta-analysis provided no evidence that healthy individual preferentially activates the right ATL when processing social concepts. This suggests that there may be other factors influencing the association of abnormal social behaviors with right ATL damage. For example, patients with right ATL damage might have a more severe level of impairment generally or they may tend to have greater damage to other brain areas, such as orbitofrontal cortex.

The chief advantage of the meta-analysis approach is that it identifies results that are consistent across a large number of studies and is therefore resistant to weakness that are associated a particular study. It remains susceptible, however, to weaknesses that are systematic across many studies. The omission of the ventral ATL is one such systematic weakness. As described earlier, the ventral ATL has been poorly sampled in many previous neuroimaging studies due to various technical limitations, and this may disproportionately affect studies investigating visual processing [see Devlin et al. (2000) and Visser, Embleton, et al. (2010)]. For this reason, it has not appeared in this and other meta-analyses of semantic processing (Binder et al. 2009; Visser, Jefferies, et al. 2010). In contrast, PET and fMRI studies that have avoided these technical problems have reliably observed considerable semantically related, multimodal activations in ventrolateral ATL regions (with peaks centered on the anterior fusiform/ITG; Sharp et al. 2004; Binney et al. 2010; Visser and Lambon Ralph 2011; Visser et al. 2012; Hoffman et al. 2015). In addition, the same region has recently been shown to code semantic structure using multivoxel pattern analysis (Peelen and Caramazza 2012) and is the peak region when correlating the degree of semantic impairment in SD patients against the distribution of FDG hypometabolism (Mion et al. 2010). For this reason, we have included the ventral and lateral ATL in the representational hub in Figure 6 (white).

## Supplementary material

Supplementary material can be found at http://www.cercor.oxfordjournals.org/ online.

## Funding

G.E.R. was supported by a PhD studentship from EPSRC and a President's Doctoral Scholarship from the University of Manchester. The research was supported by an MRC Programme Grant (MR/J004146/1) to M.A.L.R., a Manchester Mental Health Social Care Trust Fellowship to P.H., and a Wellcome Trust Institutional Strategic Support Fund (ISSF) award (097820) to the University of Manchester. Funding to pay the Open Access publication charges for this article was provided by an RCUK block grant to the University of Manchester.

## Supplementary Material

Supplementary Data

## References

[BHV024C1] Acres K Taylor KI Moss HE Stamatakis EA Tyler LK 2009 Complementary hemispheric asymmetries in object naming and recognition: a voxel-based correlational study. Neuropsychologia. 47:1836–1843. 19428415 10.1016/j.neuropsychologia.2009.02.024

[BHV024C2] Badre D Wagner AD 2002 Semantic retrieval, mnemonic control, and prefrontal cortex. Behav Cogn Neurosci Rev. 1:206–218. 17715593 10.1177/1534582302001003002

[BHV024C4] Behrmann M Plaut DC 2013 Distributed circuits, not circumscribed centers, mediate visual recognition. Trends Cogn Sci. 17:210–219. 23608364 10.1016/j.tics.2013.03.007

[BHV024C3] Behrmann M Plaut DC 2014 Bilateral hemispheric processing of words and faces: evidence from word impairments in prosopagnosia and faces impairments in pure alexia. Cereb Cortex. 24 (4):1102–1118. 23250954 10.1093/cercor/bhs390

[BHV024C5] Bethmann A Scheich H Brechmann A 2012 The temporal lobes differentiate between the voices of famous and unknown people: an event-related fMRI study on speaker recognition. PLoS ONE. 7:15. 10.1371/journal.pone.0047626 PMC3480405 23112826

[BHV024C6] Bi Y Wei T Wu C Han Z Jiang T Caramazza A 2011 The role of the left anterior temporal lobe in language processing revisited: evidence from an individual with ATL resection. Cortex. 47:575–587. 20074721 10.1016/j.cortex.2009.12.002

[BHV024C7] Binder JR Desai RH Graves WW Conant LL 2009 Where is the semantic system? A critical review and meta-analysis of 120 functional neuroimaging studies. Cereb Cortex. 19:2767–2796. 19329570 10.1093/cercor/bhp055 PMC2774390

[BHV024C8] Binney RJ Embleton KV Jefferies E Parker GJ Lambon Ralph MA 2010 The ventral and inferolateral aspects of the anterior temporal lobe are crucial in semantic memory: evidence from a novel direct comparison of distortion-corrected fMRI, rTMS, and semantic dementia. Cereb Cortex. 20:2728–2738. 20190005 10.1093/cercor/bhq019

[BHV024C9] Binney RJ Parker GJ Lambon Ralph MA 2012 Convergent connectivity and graded specialization in the rostral human temporal lobe as revealed by diffusion-weighted imaging probabilistic tractography. J Cogn Neurosci. 24:1998–2014. 22721379 10.1162/jocn_a_00263

[BHV024C10] Blaizot X Mansilla F Insausti AM Constans JM Salinas-Alaman A Pro-Sistiaga P Mohedano-Moriano A Insausti R 2010 The Human parahippocampal region: I. Temporal pole cytoarchitectonic and MRI correlation. Cereb Cortex. 20:2198–2212. 20064939 10.1093/cercor/bhp289 PMC2923216

[BHV024C11] Blank S Scott SK Murphy K Warburton E Wise RJS 2002 Speech production: Wernicke, Broca and beyond. Brain. 125:1829–1838. 12135973 10.1093/brain/awf191

[BHV024C12] Bozeat S Lambon Ralph MA Patterson K Garrard P Hodges JR 2000 Non-verbal semantic impairment in semantic dementia. Neuropsychologia. 38:1207–1215. 10865096 10.1016/s0028-3932(00)00034-8

[BHV024C13] Brown S Schafer EA 1888 An investigation into the functions of the occipital and temporal lobes of the monkey's brain. Philos Trans R Soc Lond B Biol Sci. 179:303–327.

[BHV024C14] Butler CR Brambati SM Miller BL Gorno-Tempini ML 2009 The neural correlates of verbal and nonverbal semantic processing deficits in neurodegenerative disease. Cogn Behav Neurol. 22:73–80. 19506422 10.1097/WNN.0b013e318197925d PMC2754058

[BHV024C15] Catani M Allin MPG Husain M Pugliese L Mesulam MM Murray RM Jones DK 2007 Symmetries in human brain language pathways correlate with verbal recall. Proc Natl Acad Sci USA. 104:17163–17168. 17939998 10.1073/pnas.0702116104 PMC2040413

[BHV024C16] Chan D Anderson V Pijnenburg Y Whitwell J Barnes J Scahill R Stevens JM Barkhof F Scheltens P Rossor MN 2009 The clinical profile of right temporal lobe atrophy. Brain. 132:1287–1298. 19297506 10.1093/brain/awp037

[BHV024C17] Cohen L Lehericy S Chochon F Lemer C Rivaud S Dehaene S 2002 Language-specific tuning of visual cortex functional properties of the visual word form area. Brain. 125:1054–1069. 11960895 10.1093/brain/awf094

[BHV024C18] Damasio H Grabowski TJ Tranel D Ponto LLB Hichwa RD Damasio AR 2001 Neural correlates of naming actions and of naming spatial relations. Neuroimage. 13:1053–1064. 11352611 10.1006/nimg.2001.0775

[BHV024C19] Damasio H Tranel D Grabowski T Adolphs R Damasio A 2004 Neural systems behind word and concept retrieval. Cognition. 92:179–229. 15037130 10.1016/j.cognition.2002.07.001

[BHV024C20] Devlin JT Russell RP Davis MH Price CJ Wilson J Moss HE Matthews PM Tyler LK 2000 Susceptibility-induced loss of signal: comparing PET and fMRI on a semantic task. Neuroimage. 11 (6 Pt 1):589–600. 10860788 10.1006/nimg.2000.0595

[BHV024C21] Ding SL Van Hoesen GW Cassell MD Poremba A 2009 Parcellation of human temporal polar cortex: a combined analysis of multiple cytoarchitectonic, chemoarchitectonic, and pathological markers. J Comput Neurol. 514:595–623. 10.1002/cne.22053 PMC3665344 19363802

[BHV024C22] Drane DL Ojemann GA Aylward E Ojemann JG Johnson LC Silbergeld DL Miller JW Tranel D 2008 Category-specific naming and recognition deficits in temporal lobe epilepsy surgical patients. Neuropsychologia. 46:1242–1255. 18206185 10.1016/j.neuropsychologia.2007.11.034 PMC2474808

[BHV024C23] Drane DL Ojemann JG Phatak V Loring DW Gross RE Hebb AO Silbergeld DL Miller JW Voets NL Saindane AM 2013 Famous face identification in temporal lobe epilepsy: support for a multimodal integration model of semantic memory. Cortex. 49 (6):1648–1667. 23040175 10.1016/j.cortex.2012.08.009 PMC3679345

[BHV024C24] Duffau H 2005 Lessons from brain mapping in surgery for low-grade glioma: insights into associations between tumour and brain plasticity. Lancet Neurol. 4:476–486. 16033690 10.1016/S1474-4422(05)70140-X

[BHV024C25] Dundas EM Plaut DC Behrmann M 2013 The joint development of hemispheric lateralization for words and faces. J Exp Psychol Gen. 142:348–358. 22866684 10.1037/a0029503 PMC4241688

[BHV024C26] Edwards-Lee T Miller BL Benson DF Cummings JL Russell GL Boone K Mena I 1997 The temporal variant of frontotemporal dementia. Brain. 120 (Pt 6):1027–1040. 9217686 10.1093/brain/120.6.1027

[BHV024C27] Eickhoff SB Laird AR Grefkes C Wang LE Zilles K Fox PT 2009 Coordinate-based activation likelihood estimation meta-analysis of neuroimaging data: a random-effects approach based on empirical estimates of spatial uncertainty. Hum Brain Mapp. 30:2907–2926. 19172646 10.1002/hbm.20718 PMC2872071

[BHV024C28] Elfgren C van Westen D Passant U Larsson EM Mannfolk P Fransson P 2006 fMRI activity in the medial temporal lobe during famous face processing. Neuroimage. 30:609–616. 16275141 10.1016/j.neuroimage.2005.09.060

[BHV024C29] Ellis AW Young AW Critchley EM 1989 Loss of memory for people following temporal lobe damage. Brain. 112:1469–1483. 2597991 10.1093/brain/112.6.1469

[BHV024C30] Evans JJ Heggs AJ Antoun N Hodges JR 1995 Progressive prosopagnosia associated with selective right temporal lobe atrophy. A new syndrome? Brain. 118 (Pt 1):1–13. 7894996 10.1093/brain/118.1.1

[BHV024C31] Frith U Frith CD 2003 Development and neurophysiology of mentalizing. Philos Trans R Soc Lond B Biol Sci. 358:459–473. 12689373 10.1098/rstb.2002.1218 PMC1693139

[BHV024C32] Gainotti G 2007 Different patterns of famous people recognition disorders in patients with right and left anterior temporal lesions: a systematic review. Neuropsychologia. 45:1591–1607. 17275042 10.1016/j.neuropsychologia.2006.12.013

[BHV024C33] Gainotti G 2012 The format of conceptual representations disrupted in semantic dementia: a position paper. Cortex. 48:521–529. 21807363 10.1016/j.cortex.2011.06.019

[BHV024C34] Gainotti G 2013 Laterality effects in normal subjects’ recognition of familiar faces, voices and names. Perceptual and representational components. Neuropsychologia. 51:1151–1160. 23542500 10.1016/j.neuropsychologia.2013.03.009

[BHV024C35] Gainotti G Barbier A Marra C 2003 Slowly progressive defect in recognition of familiar people in a patient with right anterior temporal atrophy. Brain. 126 (Pt 4):792–803. 12615639 10.1093/brain/awg092

[BHV024C36] Gallate J Wong C Ellwood S Chi R Snyder A 2011 Noninvasive brain stimulation reduces prejudice scores on an implicit association test. Neuropsychology. 25:185–192. 21381825 10.1037/a0021102

[BHV024C37] Galton CJ Patterson K Graham K Lambon-Ralph MA Williams G Antoun N Sahakian BJ Hodges JR 2001 Differing patterns of temporal atrophy in Alzheimer's disease and semantic dementia. Neurology. 57:216–225. 11468305 10.1212/wnl.57.2.216

[BHV024C38] Gloor P 1997 The temporal lobe and the limbic system. New York: Oxford University Press.

[BHV024C39] Glosser G Salvucci AE Chiaravalloti ND 2003 Naming and recognizing famous faces in temporal lobe epilepsy. Neurology. 61:81–86. 12847161 10.1212/01.wnl.0000073621.18013.e1

[BHV024C40] Gorno-Tempini ML Price CJ 2001 Identification of famous faces and buildings: a functional neuroimaging study of semantically unique items. Brain. 124 (Pt 10):2087–2097. 11571224 10.1093/brain/124.10.2087

[BHV024C41] Grabowski TJ Damasio H Tranel D Ponto LL Hichwa RD Damasio AR 2001 A role for left temporal pole in the retrieval of words for unique entities. Hum Brain Mapp. 13:199–212. 11410949 10.1002/hbm.1033 PMC6871982

[BHV024C42] Hasan KM Iftikhar A Kamali A Kramer LA Ashtari M Cirino PT Papanicolaou AC Fletcher JM Ewing-Cobbs L 2009 Development and aging of the healthy human brain uncinate fasciculus across the lifespan using diffusion tensor tractography. Brain Res. 1276:67–76. 19393229 10.1016/j.brainres.2009.04.025 PMC2693464

[BHV024C43] Highley JR Walker MA Esiri MM Crow TJ Harrison PJ 2002 Asymmetry of the uncinate fasciculus: a post-mortem study of normal subjects and patients with schizophrenia. Cereb Cortex. 12:1218–1224. 12379610 10.1093/cercor/12.11.1218

[BHV024C44] Hodges JR Patterson K Oxbury S Funnell E 1992 Semantic dementia—progressive fluent aphasia with temporal-lobe atrophy. Brain. 115:1783–1806. 1486461 10.1093/brain/115.6.1783

[BHV024C45] Hoffman P Binney RJ Lambon Ralph MA 2015 Differing contributions of inferior prefrontal and anterior temporal cortex to concrete and abstract conceptual knowledge. Cortex. 63:250–266. 25303272 10.1016/j.cortex.2014.09.001 PMC4317194

[BHV024C46] Jefferies E 2013 The neural basis of semantic cognition: converging evidence from neuropsychology, neuroimaging and TMS. Cortex. 49:611–625. 23260615 10.1016/j.cortex.2012.10.008

[BHV024C47] Jefferies E Lambon Ralph MA 2006 Semantic impairment in stroke aphasia versus semantic dementia: a case-series comparison. Brain. 129 (Pt 8):2132–2147. 16815878 10.1093/brain/awl153

[BHV024C48] Jefferies E Patterson K Jones RW Lambon Ralph MA 2009 Comprehension of concrete and abstract words in semantic dementia. Neuropsychology. 23:492–499. 19586212 10.1037/a0015452 PMC2801065

[BHV024C49] Keidel JL Welbourne SR Lambon Ralph MA 2010 Solving the paradox of the equipotential and modular brain: a neurocomputational model of stroke vs. slow-growing glioma. Neuropsychologia. 48:1716–1724. 20188115 10.1016/j.neuropsychologia.2010.02.019

[BHV024C50] Kluver H Bucy PC 1939 Preliminary analysis of functions of the temporal lobes in monkeys. Arch Neurol Psychiatry. 42:979–1000. 10.1176/jnp.9.4.606 9447506

[BHV024C51] Kluver H Bucy PC 1937 “Psychic blindness” and other symptoms following bilateral temporal lobectomy in rhesus monkeys. Am J Physiol. 119:352.

[BHV024C52] Kubicki M Westin CF Maier SE Frumin M Nestor PG Salisbury DF Kikinis R Jolesz FA McCarley RW Shenton ME 2002 Uncinate fasciculus findings in schizophrenia: a magnetic resonance diffusion tensor imaging study. Am J Psychiatry. 159:813–820. 11986136 10.1176/appi.ajp.159.5.813 PMC2803760

[BHV024C53] Laird AR Fox PM Price CJ Glahn DC Uecker AM Lancaster JL Turkeltaub PE Kochunov P Fox PT 2005 ALE meta-analysis: controlling the false discovery rate and performing statistical contrasts. Hum Brain Mapp. 25:155–164. 15846811 10.1002/hbm.20136 PMC6871747

[BHV024C54] Lambon Ralph MA 2014 Neurocognitive insights on conceptual knowledge and its breakdown. Philos Trans R Soc Lond B Biol Sci. 369:1–12. 10.1098/rstb.2012.0392 PMC3866422 24324236

[BHV024C55] Lambon Ralph MA Cipolotti L Manes F Patterson K 2010 Taking both sides: do unilateral anterior temporal lobe lesions disrupt semantic memory? Brain. 133:3243–3255. 20952378 10.1093/brain/awq264

[BHV024C56] Lambon Ralph MA Ehsan S Baker GA Rogers TT 2012 Semantic memory is impaired in patients with unilateral anterior temporal lobe resection for temporal lobe epilepsy. Brain. 135 (Pt 1):242–258. 22287382 10.1093/brain/awr325 PMC3267985

[BHV024C57] Lambon Ralph MA McClelland JL Patterson K Galton CJ Hodges JR 2001 No right to speak? The relationship between object naming and semantic impairment: neuropsychological evidence and a computational model. J Cogn Neurosci. 13:341–356. 11371312 10.1162/08989290151137395

[BHV024C58] Lambon Ralph MA Pobric G Jefferies E 2009 Conceptual knowledge is underpinned by the temporal pole bilaterally: convergent evidence from rTMS. Cereb Cortex. 19:832–838. 18678765 10.1093/cercor/bhn131

[BHV024C59] Lambon Ralph MA Sage K Jones RW Mayberry EJ 2010 Coherent concepts are computed in the anterior temporal lobes. Proc Natl Acad Sci USA. 107:2717–2722. 20133780 10.1073/pnas.0907307107 PMC2823909

[BHV024C60] Marinkovic K Dhond RP Dale AM Glessner M Carr V Halgren E 2003 Spatiotemporal dynamics of modality-specific and supramodal word processing. Neuron. 38:487–497. 12741994 10.1016/s0896-6273(03)00197-1 PMC3746792

[BHV024C61] Mesulam MM 1998 From sensation to cognition. Brain. 121:1013–1052. 9648540 10.1093/brain/121.6.1013

[BHV024C62] Mesulam MM Wieneke C Hurley R Rademaker A Thompson CK Weintraub S Rogalski EJ 2013 Words and objects at the tip of the left temporal lobe in primary progressive aphasia. Brain. 136:601–618. 23361063 10.1093/brain/aws336 PMC3572925

[BHV024C63] Miller BL Darby A Benson DF Cummings JL Miller MH 1997 Aggressive, socially disruptive and antisocial behaviour associated with fronto-temporal dementia. Br J Psychiatry. 170:150–155. 9093504 10.1192/bjp.170.2.150

[BHV024C64] Mion M Patterson K Acosta-Cabronero J Pengas G Izquierdo-Garcia D Hong YT Fryer TD Williams GB Hodges JR Nestor PJ 2010 What the left and right anterior fusiform gyri tell us about semantic memory. Brain. 133:3256–3268. 20952377 10.1093/brain/awq272

[BHV024C65] Moll J Zahn R de Oliveira-Souza R Krueger F Grafman J 2005 The neural basis of human moral cognition. Nat Rev Neurosci. 6:799–809. 16276356 10.1038/nrn1768

[BHV024C66] Moran MA Mufson EJ Mesulam MM 1987 Neural inputs into the temporopolar cortex of the rhesus monkey. J Comp Neurol. 256:88–103. 3819040 10.1002/cne.902560108

[BHV024C67] Mummery CJ Shallice T Price CJ 1999 Dual-process model in semantic priming: a functional imaging perspective. Neuroimage. 9:516–525. 10329291 10.1006/nimg.1999.0434

[BHV024C68] Olson IR McCoy D Klobusicky E Ross LA 2013 Social cognition and the anterior temporal lobes: a review and theoretical framework. Soc Cogn Affect Neurosci. 8:123–133. 23051902 10.1093/scan/nss119 PMC3575728

[BHV024C69] Olson IR Ploaker A Ezzyat Y 2007 The enigmatic temporal pole: a review of findings on social and emotional processing. Brain. 130:1718–1731. 17392317 10.1093/brain/awm052

[BHV024C70] Parker GJM Luzzi S Alexander DC Wheeler-Kingshott CAM Clecarelli O Lambon Ralph MA 2005 Lateralization of ventral and dorsal auditory-language pathways in the human brain. Neuroimage. 24:656–666. 15652301 10.1016/j.neuroimage.2004.08.047

[BHV024C71] Pascual B Masdeu JC Hollenbeck M Makris N Insausti R Ding SL Dickerson BC 2015 Large-scale brain networks of the human left temporal pole: a functional connectivity MRI study. Cereb Cortex. 25 (3):680–702. 24068551 10.1093/cercor/bht260 PMC4318532

[BHV024C72] Patterson K Nestor PJ Rogers TT 2007 Where do you know what you know? The representation of semantic knowledge in the human brain. Nat Rev Neurosci. 8:976–987. 18026167 10.1038/nrn2277

[BHV024C73] Peelen MV Caramazza A 2012 Conceptual object representations in human anterior temporal cortex. J Neurosci. 32:15728–15736. 23136412 10.1523/JNEUROSCI.1953-12.2012 PMC6621609

[BHV024C74] Plaut DC 2002 Graded modality-specific specialisation in semantics: a computational account of optic aphasia. Cogn Neuropsychol. 19:603–639. 20957556 10.1080/02643290244000112

[BHV024C75] Plaut DC Behrmann M 2011 Complementary neural representations for faces and words: a computational exploration. Cogn Neuropsychol. 28:251–275. 22185237 10.1080/02643294.2011.609812

[BHV024C76] Pobric G Jefferies E Lambon Ralph MA 2010 Amodal semantic representations depend on both anterior temporal lobes: evidence from repetitive transcranial magnetic stimulation. Neuropsychologia. 48:1336–1342. 20038436 10.1016/j.neuropsychologia.2009.12.036

[BHV024C77] Pobric G Jefferies E Lambon Ralph MA 2007 Anterior temporal lobes mediate semantic representation: mimicking semantic dementia by using rTMS in normal participants. Proc Natl Acad Sci. 104:20137–20141. 18056637 10.1073/pnas.0707383104 PMC2148435

[BHV024C78] Reiman EM Lane RD Ahern GL Schwartz GE Davidson RJ Friston KJ Yun LS Chen KW 1997 Neuroanatomical correlates of externally and internally generated human emotion. Am J Psychiatry. 154:918–925. 9210741 10.1176/ajp.154.7.918

[BHV024C79] Rogers TT Lambon Ralph MA Garrard P Bozeat S McClelland JL Hodges JR Patterson K 2004 Structure and deterioration of semantic memory: a neuropsychological and computational investigation. Psychol Rev. 111:205–235. 14756594 10.1037/0033-295X.111.1.205

[BHV024C80] Ross LA Olson IR 2010 Social cognition and the anterior temporal lobes. Neuroimage. 49:3452–3462. 19931397 10.1016/j.neuroimage.2009.11.012 PMC2818559

[BHV024C81] Ross LA Olson IR 2012 What's unique about unique entities? An fMRI investigation of the semantics of famous faces and landmarks. Cereb Cortex. 22:2005–2015. 22021913 10.1093/cercor/bhr274 PMC3412440

[BHV024C82] Schapiro AC McClelland JL Welbourne SR Rogers TT Lambon Ralph MA 2013 Why bilateral damage is worse than unilateral damage to the brain. J Cogn Neurosci. 25:2107–2123. 23806177 10.1162/jocn_a_00441

[BHV024C83] Schmahmann JD Pandya DN Wang R Dai G D'Arceuil HE de Crespigny AJ Wedeen VJ 2007 Association fibre pathways of the brain: parallel observations from diffusion spectrum imaging and autoradiography. Brain. 130:630–653. 17293361 10.1093/brain/awl359

[BHV024C84] Scoville WB Milner B 1957 Loss of recent memory after bilateral hippocampal lesions. J Neurol Neurosurg Psychiatry. 20:11–21. 13406589 10.1136/jnnp.20.1.11 PMC497229

[BHV024C85] Seidenberg M Griffith R Sabsevitz D Moran M Haltiner A Bell B Swanson S Hammeke T Hermann B 2002 Recognition and identification of famous faces in patients with unilateral temporal lobe epilepsy. Neuropsychologia. 40:446–456. 11684177 10.1016/s0028-3932(01)00096-3

[BHV024C86] Sergent J Ohta S MacDonald B 1992 Functional neuroanatomy of face and object processing. A Positron Emission Tomography Study. Brain. 115 (Pt 1):15–36. 1559150 10.1093/brain/115.1.15

[BHV024C87] Sharp DJ Scott SK Wise RJS 2004 Retrieving meaning after temporal lobe infarction: the role of the basal language area. Ann Neurol. 56:836–846. 15514975 10.1002/ana.20294

[BHV024C88] Simmons WK Reddish M Bellgowan PS Martin A 2010 The selectivity and functional connectivity of the anterior temporal lobes. Cereb Cortex. 20:813–825. 19620621 10.1093/cercor/bhp149 PMC2837089

[BHV024C89] Skipper LM Ross LA Olson IR 2011 Sensory and semantic category subdivisions within the anterior temporal lobes. Neuropsychologia. 49:3419–3429. 21889520 10.1016/j.neuropsychologia.2011.07.033 PMC3192293

[BHV024C90] Snowden JS Thompson JC Neary D 2012 Famous people knowledge and the right and left temporal lobes. Behav Neurol. 25:35–44. 22207421 10.3233/BEN-2012-0347 PMC5294234

[BHV024C91] Snowden JS Thompson JC Neary D 2004 Knowledge of famous faces and names in semantic dementia. Brain. 127 (Pt 4):860–872. 14985259 10.1093/brain/awh099

[BHV024C92] Spitsyna G Warren JE Scott SK Turkheimer FE Wise RJS 2006 Converging language streams in the human temporal lobe. J Neurosci. 26:7328–7336. 16837579 10.1523/JNEUROSCI.0559-06.2006 PMC6674192

[BHV024C93] Terzian H Ore GD 1955 Syndrome of Kluver and Bucy—Reproduced in man by bilateral removal of the temporal lobes. Neurology. 5:373–380. 14383941 10.1212/wnl.5.6.373

[BHV024C94] Thiebaut de Schotten M Ffytche DH Bizzi A Dell'Acqua F Allin M Walshe M Murray R Williams SC Murphy DG Catani M 2011 Atlasing location, asymmetry and inter-subject variability of white matter tracts in the human brain with MR diffusion tractography. Neuroimage. 54:49–59. 20682348 10.1016/j.neuroimage.2010.07.055

[BHV024C95] Thompson SA Patterson K Hodges JR 2003 Left/right asymmetry of atrophy in semantic dementia: behavioral-cognitive implications. Neurology. 61:1196–1203. 14610120 10.1212/01.wnl.0000091868.28557.b8

[BHV024C96] Thompson-Schill SL D'Esposito M Aguirre GK Farah MJ 1997 Role of left inferior prefrontal cortex in retrieval of semantic knowledge: A reevaluation. Proc Natl Acad Sci USA. 94:14792–14797. 9405692 10.1073/pnas.94.26.14792 PMC25116

[BHV024C97] Tranel D Damasio H Damasio AR 1997 A neural basis for the retrieval of conceptual knowledge. Neuropsychologia. 35:1319–1327. 9347478 10.1016/s0028-3932(97)00085-7

[BHV024C98] Tsapkini K Frangakis CE Hillis AE 2011 The function of the left anterior temporal pole: evidence from acute stroke and infarct volume. Brain. 134:3094–3105. 21685458 10.1093/brain/awr050 PMC3187536

[BHV024C99] Tsukiura T Mano Y Sekiguchi A Yomogida Y Hoshi K Kambara T Takeuchi H Sugiura M Kawashima R 2010 Dissociable roles of the anterior temporal regions in successful encoding of memory for person identity information. J Cogn Neurosci. 22:2226–2237. 19803684 10.1162/jocn.2009.21349

[BHV024C100] Tsukiura T Mochizuki-Kawai H Fujii T 2006 Dissociable roles of the bilateral anterior temporal lobe in face-name associations: an event-related fMRI study. Neuroimage. 30:617–626. 16275140 10.1016/j.neuroimage.2005.09.043

[BHV024C101] Tsukiura T Suzuki C Shigemune Y Mochizuki-Kawai H 2008 Differential contributions of the anterior temporal and medial temporal lobe to the retrieval of memory for person identity information. Hum Brain Mapp. 29:1343–1354. 17948885 10.1002/hbm.20469 PMC6870856

[BHV024C102] Turkeltaub PE Coslett HB 2010 Localization of sublexical speech perception components. Brain Lang. 114:1–15. 20413149 10.1016/j.bandl.2010.03.008 PMC2914564

[BHV024C103] Vandenberghe R Nobre AC Price CJ 2002 The response of left temporal cortex to sentences. J Cogn Neurosci. 14:550–560. 12126497 10.1162/08989290260045800

[BHV024C104] Vigliocco G Kousta ST Della Rosa PA Vinson DP Tettamanti M Devlin JT Cappa SF 2014 The neural representation of abstract words: The role of emotion. Cereb Cortex. 24 (7):1767–1777. 23408565 10.1093/cercor/bht025

[BHV024C105] Visser M Embleton KV Jefferies E Parker GJ Lambon Ralph MA 2010 The inferior, anterior temporal lobes and semantic memory clarified: Novel evidence from distortion-corrected fMRI. Neuropsychologia. 48:1689–1696. 20176043 10.1016/j.neuropsychologia.2010.02.016

[BHV024C106] Visser M Jefferies E Embleton KV Lambon Ralph MA 2012 Both the middle temporal gyrus and the ventral anterior temporal area are crucial for multimodal semantic processing: distortion-corrected fMRI evidence for a double gradient of information convergence in the temporal lobes. J Cogn Neurosci. 24:1766–1778. 22621260 10.1162/jocn_a_00244

[BHV024C107] Visser M Jefferies E Lambon Ralph MA 2010 Semantic processing in the anterior temporal lobes: a meta-analysis of the functional neuroimaging literature. J Cogn Neurosci. 22:1083–1094. 19583477 10.1162/jocn.2009.21309

[BHV024C108] Visser M Lambon Ralph MA 2011 Differential contributions of bilateral ventral anterior temporal lobe and left anterior superior temporal gyrus to semantic processes. J Cogn Neurosci. 23:3121–3131. 21391767 10.1162/jocn_a_00007

[BHV024C109] Von Der Heide R Skipper L Olson IR 2013 Anterior temporal face patches: a meta-analysis and empirical study. Front Hum Neurosci. 7:18. 23378834 10.3389/fnhum.2013.00017 PMC3561664

[BHV024C110] Von Der Heide RJ Skipper LM Klobusicky E Olson IR 2013 Dissecting the uncinate fasciculus: disorders, controversies and a hypothesis. Brain. 136:1692–1707. 23649697 10.1093/brain/awt094 PMC3673595

[BHV024C111] Wong C Gallate J 2012 The function of the anterior temporal lobe: a review of the empirical evidence. Brain Res. 1449:94–116. 22421014 10.1016/j.brainres.2012.02.017

[BHV024C112] Zahn R Moll J Iyengar V Huey ED Tierney M Krueger F Grafman J 2009 Social conceptual impairments in frontotemporal lobar degeneration with right anterior temporal hypometabolism. Brain. 132:604–616. 19153155 10.1093/brain/awn343 PMC2724922

[BHV024C113] Zahn R Moll J Krueger F Huey ED Garrido G Grafman J 2007 Social concepts are represented in the superior anterior temporal cortex. Proc Natl Acad Sci USA. 104:6430–6435. 17404215 10.1073/pnas.0607061104 PMC1851074

