# Supplementary material for: The Roles of Left Versus Right Anterior Temporal Lobes in Conceptual Knowledge: An ALE Meta-analysis of 97 Functional Neuroimaging Studies
Source: Cereb Cortex. 2015 Mar 13;25(11):4374–91. doi: 10.1093/cercor/bhv024 (PMC4816787; doi:10.1093/cercor/bhv024)
Supplement: Supplementary Data [file supp_25_11_4374__index.html]

The Roles of Left Versus Right Anterior Temporal Lobes in Conceptual Knowledge: An ALE Meta-analysis of 97 Functional Neuroimaging Studies — Supplementary Data 

# The Roles of Left Versus Right Anterior Temporal Lobes in Conceptual Knowledge: An ALE Meta-analysis of 97 Functional Neuroimaging Studies

## Supplementary Data

Supplementary Data

**Files in this Data Supplement:**

- Supplementary Data - Docx file
