# Supplementary material for: The Roles of Left Versus Right Anterior Temporal Lobes in Conceptual Knowledge: An ALE Meta-analysis of 97 Functional Neuroimaging Studies
Source: Cereb Cortex. 2015 Mar 13;25(11):4374–91. doi: 10.1093/cercor/bhv024 (PMC4816787; doi:10.1093/cercor/bhv024)
Supplement: Supplementary Data [file supp_bhv024_bhv024supp.docx]

## Supplementary materials

**Supplementary Figure 1:** activation likelihood maps for the different study sub-types within (A) the sensory input analysis, (B) the word retrieval task analysis and (C) the social semantic category analysis.

**
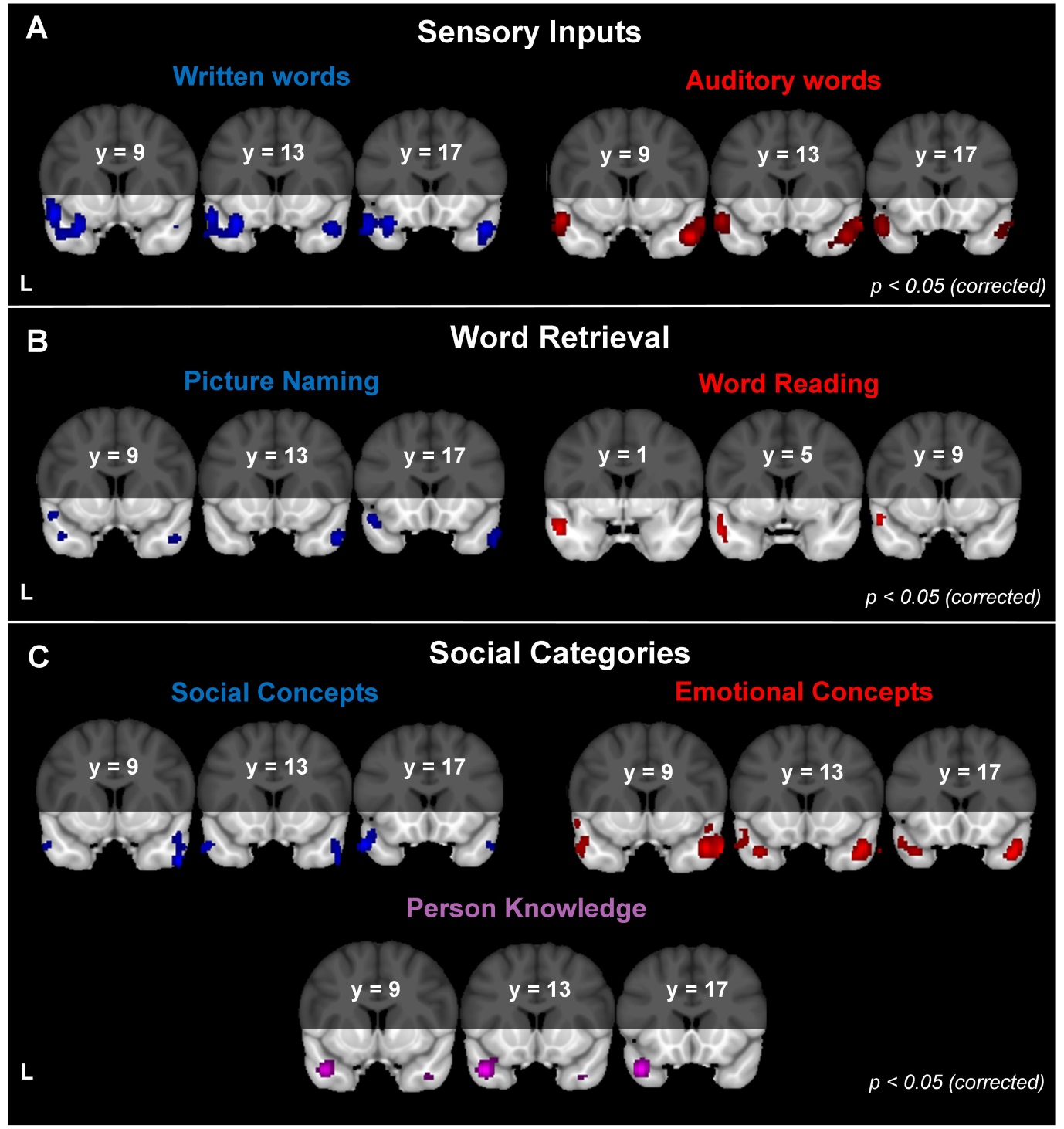
**

Supplementary Table 1: list of the 97 neuroimaging studies included in the meta-analysis, coded by study type.

| **Study** | **Input Modality** | **Sensory Input** | **Word Retrieval** | **Speech Output** | **Semantic Category** | **Social category** |
| --- | --- | --- | --- | --- | --- | --- |
| Aalto et al (2002) | Non-Verbal | Visual | Non-Verbal | NA | Social | Emotion |
| Barenese et al (2011) | Non-Verbal | Visual | Non-Verbal | NA | Social | People |
| Blair et al (1999) | Non-Verbal | Visual | Non-Verbal | NA | Social | Emotion |
| Bottini et al (1994) | Verbal | Written | Verbal | Reading | Non Social | NA |
| Brambati et al (2010) | Non-Verbal | Visual | Non-Verbal | NA | Social | People |
| Bright et al (2004) | Verbal | Written | Non-Verbal | NA | Non-Social | NA |
| Bright et al (2004) | Non-Verbal | Visual | Non-Verbal | NA | Non-Social | NA |
| Brunet et al (2000) | Non-Verbal | Visual | Non-Verbal | NA | Social | Concepts |
| Cai et al (2007) | Verbal | Auditory | Non-Verbal | NA | Non Social | NA |
| Crinion et al (2003) | Verbal | Auditory | Non-Verbal | NA | Non Social | NA |
| Damasio et al (2001) | Non-Verbal | Visual | Verbal | Naming | Non Social | NA |
| Damasio et al (2004) | Non-Verbal | Visual | Verbal | Naming | Social | People |
| Dapretto et al (1999) | Verbal | Auditory | Non-Verbal | NA | Non-Social | NA |
| D'Arcy et al (2007) | None | NA | Non-Verbal | NA | Non-Social | NA |
| Den Outen et al (2005) | Verbal | Written | Non-Verbal | NA | Social | Concepts |
| Devlin et al (2000) | Verbal | Written | Non-Verbal | NA | Non-Social | NA |
| Devlin et al (2002) | Verbal | Written | Non-Verbal | NA | Non-Social | NA |
| Dolan et al (2000) | Non-Verbal | Visual | Non-Verbal | NA | Social | Emotion |
| Dougherty et al (1999) | Verbal | Auditory | Non-Verbal | NA | Social | Emotion |
| Elfgren et al (2006) | Non-Verbal | Visual | Verbal | Naming | Social | People |
| Engelien et al (2006) | Non-Verbal | Auditory | Non-Verbal | NA | Non Social | NA |
| Eugene et al (2003) | Non-Verbal | Visual | Non-Verbal | NA | Social | Emotion |
| Farias et al (2005) | Verbal | Auditory | Verbal | Naming | Non Social | NA |
| Ferstl et al (2005) | Verbal | Auditory | Non-Verbal | NA | Non-Social | NA |
| Fletcher et al (1995) | Verbal | Written | Verbal | Reading | Social | Concepts |
| Foki et al (2008) | Verbal | Written | Verbal | Reading | Non-Social | NA |
| Gerlach et al (1999) | Non-Verbal | Visual | Non-Verbal | NA | Non-Social | NA |
| Gesierich et al (2012) | Non-Verbal | Visual | Non-Verbal | NA | Social | People |
| Giraud et al (2004) | Verbal | Auditory | Non-Verbal | NA | Non-Social | NA |
| Gorno-Tempini et al (1998) | Verbal | Written | Non-Verbal | NA | Social | People |
| Gorno-Tempini et al (1998) | Non-Verbal | Visual | Non-Verbal | NA | Social | People |
| Grabowski et al (2001) | Non-Verbal | Visual | Verbal | Naming | Social | People |
| Heekeren et al (2003) | Verbal | Written | Non-Verbal | NA | Social | Concepts |
| Heim et al (2008) | Verbal | Written | Verbal | Naming | Non-Social | NA |
| Herbster et al (1997) | Verbal | Written | Verbal | Reading | Non-Social | NA |
| Humphries et al (2006) | Verbal | Auditory | Non-Verbal | NA | Non-Social | NA |
| Ikuta et al (2006) | Verbal | Written | Verbal | Reading | Non-Social | NA |
| Iwase et al (2002) | Non-Verbal | Visual | Non-Verbal | NA | Social | Emotion |
| Kang et al (2006) | Verbal | Auditory | Non-Verbal | NA | Non-Social | NA |
| Kimbrell et al (1999) | Non-Verbal | Visual | Non-Verbal | NA | Social | Emotion |
| Kiyosawa et al (1996) | Non-Verbal | Visual | Verbal | Naming | Non-Social | NA |
| Kohler et al (2000) | None | NA | Non-Verbal | NA | Non-Social | NA |
| Leveroni et al (2000) | Non-Verbal | Visual | Non-Verbal | NA | Social | People |
| Levesque et al (2003) | Non-Verbal | Visual | Non-Verbal | NA | Social | Emotion |
| Li et al (2004) | Verbal | Written | Non-Verbal | NA | Non-Social | NA |
| Lindenberg et al (2007) | Verbal | Written | Verbal | Reading | Non-Social | NA |
| Lindenberg et al (2007) | Verbal | Auditory | Non-Verbal | NA | Non-Social | NA |
| Moll et al (2001) | Verbal | Auditory | Non-Verbal | NA | Social | Concepts |
| Moll et al (2002)a | Verbal | Written | Non-Verbal | NA | Social | Concepts |
| Moll et al (2002)b | Non-Verbal | Visual | Non-Verbal | NA | Social | Concepts |
| Mummery et al (1998) | Verbal | Written | Non-Verbal | NA | Non-Social | NA |
| Murtha et al (1999) | Non-Verbal | Visual | Verbal | Naming | Non-Social | NA |
| Nakamura et al (2000) | Non-Verbal | Visual | Non-Verbal | NA | Social | People |
| Nakamura et al (2001) | Verbal | Auditory | Non-Verbal | NA | Social | People |
| Nielson et al (2010) | None | NA | Non-Verbal | NA | Social | People |
| Noppeney et al (2002) | Verbal | Auditory | Non-Verbal | NA | Non Social | NA |
| Noppeney et al (2004) | Verbal | Written | Non-Verbal | NA | Non-Social | NA |
| Ohira et al (2006) | Non-Verbal | Visual | Non-Verbal | NA | Social | Emotion |
| Papathianassou et al (2000) | Verbal | Auditory | Verbal | Naming | Non Social | NA |
| Peele et al (2004) | Verbal | Auditory | Non-Verbal | NA | Non Social | NA |
| Peelen et al (2012) | Non-Verbal | Visual | Non-Verbal | NA | Non-Social | NA |
| Pelletier et al (2003) | None | NA | None | NA | Social | Emotion |
| Perani et al (1999) | Verbal | Written | Verbal | Reading | Non-Social | NA |
| Platel et al (1997) | Non-Verbal | Auditory | Non-Verbal | NA | Non Social | NA |
| Platel et al (2003) | Non-Verbal | Auditory | Non-Verbal | NA | Non-Social | NA |
| Postler et al (2003) | Verbal | Written | Non-Verbal | NA | Non-Social | NA |
| Price et al (1996) | Verbal | Auditory | Non-Verbal | NA | Non-Social | NA |
| Price et al (2005) | Non-Verbal | Visual | Non-Verbal | NA | Non-Social | NA |
| Reiman et al (1997) | Non-Verbal | Visual | Non-Verbal | NA | Social | Emotion |
| Rodd et al (2005) | Verbal | Auditory | Non-Verbal | NA | Non-Social | NA |
| Rogers et al (2006) | Non-Verbal | Visual | Non-Verbal | NA | Non Social | NA |
| Roskies et al (2001) | Verbal | Written | Non-Verbal | NA | Non-Social | NA |
| Ross et al (2010) | Verbal | Written | Non-Verbal | NA | Social | Concepts |
| Ross et al (2010) | Non-Verbal | Visual | Non-Verbal | NA | Social | Concepts |
| Ross et al (2012) | Non-Verbal | Visual | Non-Verbal | NA | Social | People |
| Rothstein et al (2005) | Non-Verbal | Visual | Non-Verbal | NA | Social | People |
| Saxe et al (2006) | Verbal | Written | Verbal | Reading | Social | Concepts |
| Schaich Borg et al (2006) | Verbal | Written | Non-Verbal | NA | Social | Concepts |
| Scott et al (2000) | Verbal | Auditory | Non-Verbal | NA | Non-Social | NA |
| Scott et al (2004) | Verbal | Auditory | Non-Verbal | NA | Non-Social | NA |
| Sergent et al (1992) | Non-Verbal | Visual | Non-Verbal | NA | Social | People |
| Shin et al (2000) | Verbal | Auditory | Non-Verbal | NA | Social | Emotion |
| Skipper et al (2011) | None | NA | Non-Verbal | NA | Social | Concepts |
| Spunt et al (2011) | Non-Verbal | Visual | Verbal | Naming | Social | Concepts |
| Stowe et al (1999) | Verbal | Written | Verbal | Reading | Non Social | NA |
| Suigura et al (2001) | Non-Verbal | Visual | Non-Verbal | NA | Social | People |
| Suigura et al (2006) | Verbal | Written | Non-Verbal | NA | Social | People |
| Suigura et al (2008) | Verbal | Written | Non-Verbal | NA | Social | People |
| Takahashi et al (2004) | Verbal | Written | Verbal | Reading | Social | Emotion |
| Thierry et al (2006) | None | NA | Non-Verbal | NA | Non-Social | NA |
| Tieleman et al (2005) | Verbal | Written | Non-Verbal | NA | Non Social | NA |
| Tyler et al (2003) | Verbal | Written | Non-Verbal | NA | Non-Social | NA |
| Vandenberghe et al (1996) | Verbal | Written | Non-Verbal | NA | Non-Social | NA |
| Vandenberghe et al (2002) | Verbal | Written | Verbal | Reading | Non-Social | NA |
| Zahn et al (2007) | Verbal | Written | Non-Verbal | NA | Social | Concepts |
| Zahn et al (2009) | Verbal | Written | Non-Verbal | NA | Social | Concepts |

**Supplementary Table 2: Activation likelihood clusters for each study type**. Brodmann labels are given in parenthesis.

|  |  |  |  | **Peak MNI coordinates** | | |  |
| --- | --- | --- | --- | --- | --- | --- | --- |
| **Study Type** | **Cluster No.** | **Cluster size (mm^3^)** | **Peak Location (BA)** | **X** | **Y** | **Z** | **ALE value** |
| **Input modality** | | | | | | | |
| **Verbal** | 1 | 13 688 | STG (22) | -52 | 8 | -18 | 0.05 |
|  |  |  | STG (38) | -48 | 18 | -24 | 0.04 |
|  |  |  | STG (38) | -32 | 12 | -22 | 0.02 |
|  |  |  | STG (38) | -56 | 4 | -4 | 0.02 |
|  |  |  | ITG (20) | -34 | 18 | -34 | 0.02 |
|  | 2 | 10 056 | MTG (21) | 50 | 8 | -32 | 0.05 |
|  |  |  | MTG (21) | 58 | 10 | -20 | 0.03 |
|  |  |  | MTG (21) | 60 | 2 | -16 | 0.03 |
|  |  |  | Parahipp. gyrus (36) | 28 | 4 | -30 | 0.02 |
|  | 3 | 1416 | ITG (20) | -42 | -16 | -36 | 0.02 |
|  |  |  | ITG (20) | -30 | 0 | -42 | 0.02 |
| **Non-Verbal** | 1 | 12 592 | ITG (20) | -38 | 14 | -32 | 0.04 |
|  |  |  | MTG (21) | -54 | 6 | -28 | 0.03 |
|  |  |  | STG (38) | -46 | 18 | -18 | 0.02 |
|  |  |  | MTG (21) | -56 | -4 | -20 | 0.02 |
|  |  |  | Hippocampus (28) | -24 | -6 | -20 | 0.02 |
|  |  |  | Fusiform gyrus (20) | -32 | -8 | -32 | 0.02 |
|  | 2 | 7752 | MTG (21) | 60 | 2 | -18 | 0.04 |
|  |  |  | ITG (20) | 42 | 14 | -34 | 0.03 |
|  |  |  | STG (38) | 46 | 20 | -26 | 0.03 |
|  |  |  | MTG (38) | 60 | 6 | -30 | 0.02 |
|  | 3 | 904 | Parahipp. gyrus (28) | 26 | -12 | -26 | 0.02 |
|  |  |  | Amygdala (36) | 28 | 0 | -24 | 0.02 |
| **Word Retrieval** | | | | | | | |
| **Verbal** | 1 | 7656 | STG (22) | -50 | 6 | -14 | 0.02 |
|  |  |  | STG (38) | -46 | 20 | -18 | 0.02 |
|  |  |  | ITG (20) | -44 | 4 | -32 | 0.02 |
|  |  |  | MTG (21) | -52 | 0 | -24 | 0.02 |
|  |  |  | Fusiform gyrus (20) | -30 | 0 | -42 | 0.02 |
|  | 2 | 2592 | MTG (38) | 50 | 12 | -32 | 0.02 |
| **Non-Verbal** | 1 | 22 712 | STG (38) | -42 | 14 | -32 | 0.06 |
|  |  |  | STG (38) | -52 | 10 | -20 | 0.05 |
|  |  |  | MTG (21) | -56 | -6 | -18 | 0.03 |
|  |  |  | Fusiform gyrus (20) | -32 | -10 | -32 | 0.03 |
|  |  |  | ITG (20) | -44 | -14 | -34 | 0.02 |
|  |  |  | Hippocampus (28) | -24 | -6 | -20 | 0.02 |
|  |  |  | ITG (20) | -46 | -2 | -38 | 0.02 |
|  | 2 | 16 744 | MTG (21) | 60 | 2 | -18 | 0.06 |
|  |  |  | ITG (20) | 42 | 14 | -34 | 0.06 |
|  |  |  | MTG (21) | 50 | 6 | -30 | 0.05 |
|  |  |  | Parahipp. Gyrus (36) | 28 | 2 | -28 | 0.03 |
|  |  |  | Parahipp. Gyrus (36) | 26 | -12 | -26 | 0.02 |
|  |  |  | Parahipp. Gyrus (28) | 18 | -4 | -18 | 0.02 |
| **Semantic category** | | | | | | | |
| **Non-Social** | 1 | 15 776 | STG (22) | -52 | 8 | -18 | 0.06 |
|  |  |  | STG (38) | -48 | 18 | -20 | 0.04 |
|  |  |  | ITG (20) | -44 | -14 | -34 | 0.03 |
|  |  |  | ITG (20) | -34 | 8 | -32 | 0.02 |
|  |  |  | STG (38) | -32 | 18 | -34 | 0.02 |
|  |  |  | Fusiform gyrus (20) | -30 | 0 | -40 | 0.02 |
|  |  |  | STG (38) | -34 | 16 | -22 | 0.01 |
|  | 2 | 7848 | ITG (20) | 42 | 14 | -34 | 0.04 |
|  |  |  | MTG (21) | 50 | 8 | -32 | 0.03 |
|  |  |  | MTG (21) | 60 | 2 | -16 | 0.03 |
|  |  |  | MTG (21) | 60 | 10 | -20 | 0.03 |
| **Social** | 1 | 12 664 | ITG (20) | -40 | 14 | -32 | 0.05 |
|  |  |  | Amygdala (34) | -26 | -4 | -20 | 0.02 |
|  |  |  | MTG (21) | -54 | 6 | -28 | 0.02 |
|  |  |  | ITG (20) | -46 | -2 | -38 | 0.02 |
|  |  |  | MTG (21) | -56 | -4 | -18 | 0.02 |
|  |  |  | Fusiform gyrus (20) | -34 | -14 | -26 | 0.01 |
|  |  |  | Parahipp. gyrus (36) | -30 | -6 | -30 | 0.01 |
|  | 2 | 11 312 | MTG (21) | 60 | 2 | -18 | 0.04 |
|  |  |  | MTG (21) | 48 | 8 | -28 | 0.04 |
|  |  |  | STG (38) | 46 | 20 | -26 | 0.03 |
|  |  |  | MTG (21) | 60 | 6 | -30 | 0.03 |
|  | 3 | 1944 | Amygdala (36) | 28 | 2 | -26 | 0.03 |
|  |  |  | Parahipp. gyrus (36) | 26 | -12 | -26 | 0.02 |
|  |  |  | Parahipp. gyrus (28) | 18 | -4 | -18 | 0.02 |

**Supplementary Table 3:** **Activation likelihood clusters from the subtraction analyses and corresponding conjunctions for each study type**. Clusters are marked as more likely to be active in one study type compared to the other and vice versa, and regions more likely to be active in both study types (conjunction).

| **Study Type** | **Cluster No.** | **Cluster Size (mm^3^)** | **Peak location (BA)** | **Peak MNI coordinates** | | | | | **Z value** |
| --- | --- | --- | --- | --- | --- | --- | --- | --- | --- |
|  |  |  |  | **x** | | **y** | **z** | |  |
| **Input modality** | | | | | | | | | |
| **Verbal > Non-Verbal** | 1 | 1208 | STG (38) | -54 | 18 | | | -28 | 2.48 |
|  |  |  | STG (38) | -56 | 16 | | | -24 | 2.43 |
|  |  |  | STG (38) | -58 | 9 | | | -20 | 2.24 |
|  | 2 | 272 | MTG (21) | 50 | -2 | | | -34 | 2.08 |
|  | 3 | 200 | ITG (20) | -44 | -17 | | | -34 | 1.89 |
| **Non-Verbal > Verbal** | 1 | 1864 | ITG (20) | -40 | 11 | | | -28 | 2.62 |
|  |  |  | ITG (20) | -40 | 4 | | | -34 | 2.55 |
|  | 2 | 416 | Parahipp. gyrus (35) | 24 | -12 | | | -22 | 2.41 |
|  |  |  | Parahipp. gyrus (35) | 22 | -14 | | | -26 | 2.28 |
|  |  |  | Parahipp. gyrus (35) | 26 | -16 | | | -24 | 2.20 |
| **Conj: Verb. = Non-Verb.** | 1 | 6928 | MTG (21) | -54 | 6 | | | -28 | 0.03 |
|  |  |  | STG (38) | -42 | 18 | | | -26 | 0.02 |
|  |  |  | STG (38) | -46 | 18 | | | -18 | 0.02 |
|  |  |  | MTG (21) | -56 | -2 | | | -20 | 0.02 |
|  |  |  | ITG (20) | -34 | 10 | | | -30 | 0.02 |
|  |  |  | ITG (20) | -34 | 18 | | | -34 | 0.02 |
|  | 2 | 6112 | STG (38) | 44 | 14 | | | -32 | 0.03 |
|  |  |  | MTG (21) | 60 | 2 | | | -16 | 0.03 |
|  |  |  | MTG (21) | 58 | 6 | | | -30 | 0.02 |
| **Word Retrieval** | | | | | | | | | |
| **Word Retrieval > ‘Other’** | 1 | 1552 | MTG (21) | -47 | 1 | | | -20 | 2.56 |
|  |  |  | MTG (21) | -48 | 0 | | | -23 | 2.51 |
|  |  |  | ITG (20) | -46 | 4 | | | -32 | 2.38 |
|  | 2 | 976 | MTG (21) | 56 | 16 | | | -32 | 3.16 |
|  |  |  | STG (38) | 56 | 18 | | | -22 | 2.82 |
|  | 3 | 600 | STG (38) | -50 | 24 | | | -14 | 2.71 |
|  | 4 | 520 | Fusiform gyrus (36) | -34 | 2 | | | -42 | 2.45 |
|  |  |  | Fusiform gyrus (36) | -30 | 4 | | | -38 | 2.37 |
| **‘Other’ > Word Retrieval** | 1 | 4664 | MTG (21) | 66 | -6 | | | -16 | 3.54 |
|  |  |  | MTG (21) | 54 | 2 | | | -10 | 3.35 |
|  |  |  | MTG (21) | 61 | -3 | | | -15 | 3.23 |
|  |  |  | MTG (21) | 58 | 0 | | | -26 | 3.09 |
|  | 2 | 1576 | STG (38) | 38 | 21 | | | -34 | 2.77 |
| **Conj: Word Retrieval = ‘Other’** | 1 | 5760 | MTG (21) | -50 | 6 | | | -14 | 0.02 |
|  |  |  | STG (38) | -46 | 20 | | | -18 | 0.02 |
|  |  |  | ITG (20) | -44 | 6 | | | -32 | 0.02 |
|  |  |  | MTG (21) | -52 | 0 | | | -24 | 0.02 |
|  | 2 | 1856 | STG (38) | 50 | 12 | | | -32 | 0.02 |
| **Semantic category** | | | | | | | | |  |
| **Non-Social > Social** | 1 | 2536 | MTG (38) | -58 | 7 | | | -18 | 3.72 |
|  |  |  | STG (38) | -54 | 22 | | | -16 | 2.33 |
|  | 2 | 984 | ITG (20) | -48 | -14 | | | -30 | 2.49 |
|  |  |  | ITG (20) | -41 | -17 | | | -35 | 2.44 |
|  |  |  | ITG (20) | -46 | -18 | | | -33 | 2.52 |
|  | 3 | 472 | ITG (20) | -32 | 4 | | | -38 | 2.50 |
| **Social > Non-social** | 1 | 896 | Parahipp. gyrus (34) | 22 | -12 | | | -26 | 2.37 |
|  |  |  | Hippocampus (28) | 20 | -6 | | | -18 | 2.22 |
|  |  |  | Hippocampus (28) | 24 | -4 | | | -22 | 2.18 |
|  |  |  | Amygdala (20) | 30 | 0 | | | -24 | 1.91 |
|  |  |  | Parahipp. gyrus (34) | 28 | -14 | | | -24 | 1.89 |
|  | 2 | 880 | STG (38) | -40 | 16 | | | -28 | 2.73 |
|  | 3 | 416 | STG (38) | 46 | 22 | | | -18 | 2.29 |
|  | 4 | 280 | ITG (20) | 52 | 10 | | | -44 | 2.36 |
|  | 5 | 272 | Amygdala (20) | -30 | -4 | | | -18 | 1.86 |
|  |  |  | Hippocampus (28) | -24 | -4 | | | -22 | 1.84 |
| **Conj: Non-Soc. = Soc.** | 1 | 6880 | STG (38) | -46 | 18 | | | -22 | 0.03 |
|  |  |  | MTG (21) | -54 | 6 | | | -28 | 0.02 |
|  |  |  | ITG (20) | -34 | 10 | | | -32 | 0.02 |
|  |  |  | MTG (21) | -56 | -4 | | | -20 | 0.02 |
|  |  |  | ITG (20) | -32 | 16 | | | -34 | 0.02 |
|  |  |  | ITG (20) | -44 | 0 | | | -36 | 0.02 |
|  | 2 | 6720 | STG (22) | 48 | 10 | | | -30 | 0.03 |
|  |  |  | STG (38) | 44 | 16 | | | -30 | 0.03 |
|  |  |  | MTG (21) | 60 | 2 | | | -16 | 0.03 |

**Supplementary Table 4: Activation likelihood clusters from the sub-analyses within each study type.** Brodmann labels given in parentheses.

| **Study Type** | **Cluster** | **Cluster size (mm^3^)** | **Peak location (BA)** | **Peak MNI coordinates** | | | | | | | **ALE value** |
| --- | --- | --- | --- | --- | --- | --- | --- | --- | --- | --- | --- |
|  |  |  |  | **x** | | **y** | | | **z** | |  |
| **Sensory input** | | | | | | | | | | | |
| **Auditory Words** | 1 | 5896 | MTG (21) | 50 | 6 | | | -32 | | | 0.04 |
|  |  |  | MTG (21) | 58 | | | 10 | | | -20 | 0.03 |
|  |  |  | MTG (21) | 60 | | | -2 | | | -18 | 0.02 |
|  |  |  | ITG (20) | 36 | | | 12 | | | -38 | 0.01 |
|  | 2 | 4432 | STG (22) | -54 | | | 8 | | | -18 | 0.03 |
|  |  |  | STG (38) | -46 | | | 18 | | | -24 | 0.03 |
| **Written Words** | 1 | 11 320 | STG (38) | -32 | | | 12 | | | -22 | 0.02 |
|  |  |  | STG (22) | -50 | | | 8 | | | -16 | 0.02 |
|  |  |  | MTG (21) | -52 | | | -2 | | | -24 | 0.02 |
|  |  |  | STG (38) | -50 | | | 18 | | | -24 | 0.02 |
|  |  |  | STG (38) | -56 | | | 4 | | | -4 | 0.02 |
|  |  |  | ITG (20) | -44 | | | 8 | | | -32 | 0.01 |
|  | 2 | 1856 | STG (38) | 44 | | | 16 | | | -28 | 0.02 |
|  | 3 | 400 | Parahipp. gyrus (28) | 22 | | | 2 | | | -28 | 0.01 |
|  | 5 | 240 | MTG (21) | 60 | | | 4 | | | -14 | 0.01 |
| **Word Retrieval** | | | | | | | | | | | |
| **Naming** | 1 | 1304 | MTG (38) | 50 | | | 12 | | | -32 | 0.02 |
|  | 2 | 1096 | STG (38) | -46 | | | 20 | | | -18 | 0.02 |
|  | 3 | 896 | ITG (20) | -44 | | | 4 | | | -32 | 0.02 |
|  | 4 | 608 | Fusiform gyrus (20) | -30 | | | 0 | | | -42 | 0.02 |
|  | 5 | 424 | STG (38) | -50 | | | 6 | | | -14 | 0.01 |
| **Reading** | 1 | 1704 | MTG (21) | -52 | | | 2 | | | -24 | 0.01 |
|  |  |  | STG (38) | -52 | | | 8 | | | -16 | 0.01 |
|  | 2 | 240 | MTG (21) | 60 | | | 14 | | | -24 | 0.01 |
| **Semantic category** | | | | | | | | | | | |
| **Social concepts** | 1 | 2400 | ITG (20) | 52 | | | 8 | | | -42 | 0.02 |
|  |  |  | MTG (21) | 58 | | | 2 | | | -20 | 0.01 |
|  |  |  | STG (38) | 50 | | | 20 | | | -28 | 0.01 |
|  | 2 | 2104 | STG (38) | -52 | | | 16 | | | -28 | 0.02 |
|  |  |  | STG (38) | -44 | | | 20 | | | -22 | 0.01 |
|  | 3 | 200 | MTG (21) | -52 | | | 2 | | | -14 | 0.01 |
| **Person knowledge** | 1 | 3472 | ITG (20) | -42 | | | 12 | | | -32 | 0.03 |
|  |  |  | ITG (20) | -46 | | | -2 | | | -38 | 0.02 |
|  | 2 | 1320 | MTG (21) | 62 | | | 0 | | | -16 | 0.03 |
|  | 3 | 1200 | Parahipp. gyrus (36) | 28 | | | 2 | | | -18 | 0.02 |
|  |  |  | Parahipp. gyrus (36) | 26 | | | -12 | | | -26 | 0.02 |
|  | 4 | 304 | Hippocampus (35) | -22 | | | -8 | | | -18 | 0.02 |
|  | 5 | 256 | ITG (20) | 42 | | | 8 | | | -36 | 0.01 |
|  | 6 | 200 | MTG (21) | -58 | | | -6 | | | -18 | 0.02 |
| **Emotion concepts** | 1 | 5456 | STG (38) | 42 | | | 16 | | | -34 | 0.02 |
|  |  |  | ITG (20) | 46 | | | 10 | | | -28 | 0.02 |
|  |  |  | MTG (21) | 60 | | | 6 | | | -30 | 0.02 |
|  |  |  | STG (22) | 50 | | | 8 | | | -12 | 0.01 |
|  | 2 | 2864 | ITG (20) | -36 | | | 14 | | | -32 | 0.01 |
|  |  |  | MTG (21) | -54 | | | 4 | | | -28 | 0.01 |
|  |  |  | STG (38) | -46 | | | 18 | | | -26 | 0.01 |
|  | 3 | 264 | STG (38) | -56 | | | 8 | | | -8 | 0.01 |
